# Misfolded GPI-anchored proteins are escorted through the secretory pathway by ER-derived factors

**DOI:** 10.7554/eLife.46740

**Published:** 2019-05-16

**Authors:** Eszter Zavodszky, Ramanujan S Hegde

**Affiliations:** 1 MRC Laboratory of Molecular BiologyCambridgeUnited Kingdom; Weizmann InstituteIsrael; University of CambridgeUnited Kingdom

**Keywords:** protein quality control, endoplasmic reticulum, chaperone, protein trafficking, protein degradation, None

## Abstract

We have used misfolded prion protein (PrP*) as a model to investigate how mammalian cells recognize and degrade misfolded GPI-anchored proteins. While most misfolded membrane proteins are degraded by proteasomes, misfolded GPI-anchored proteins are primarily degraded in lysosomes. Quantitative flow cytometry analysis showed that at least 85% of PrP* molecules transiently access the plasma membrane *en route* to lysosomes. Unexpectedly, time-resolved quantitative proteomics revealed a remarkably invariant PrP* interactome during its trafficking from the endoplasmic reticulum (ER) to lysosomes. Hence, PrP* arrives at the plasma membrane in complex with ER-derived chaperones and cargo receptors. These interaction partners were critical for rapid endocytosis because a GPI-anchored protein induced to misfold at the cell surface was not recognized effectively for degradation. Thus, resident ER factors have post-ER itineraries that not only shield misfolded GPI-anchored proteins during their trafficking, but also provide a quality control cue at the cell surface for endocytic routing to lysosomes.

## Introduction

Maintenance of a correctly folded proteome is critical for cellular and organismal homeostasis. Consequently, all cells employ protein quality control to identify and eliminate misfolded proteins (Wolff et al., 2014). The wide diversity of proteins and the multitude of compartments in eukaryotic cells has driven the evolution of numerous quality control pathways for different classes of proteins and different types of errors. Thus, an important step in understanding the principles of cellular protein homeostasis is to delineate the recognition and degradation pathways for major classes of misfolded proteins.

Over 150 proteins in the human genome are anchored to the cell surface solely by a glycosylphosphatidylinositol (GPI) anchor in the membrane (UniProt Consortium, 2018). GPI-anchored proteins are ubiquitous across eukaryotes, often highly abundant, and have diverse roles including cell adhesion, signaling, intercellular communication, and enzymatic reactions (Kinoshita et al., 2008). How misfolded GPI-anchored proteins are selectively recognized and degraded remains poorly understood. The importance of this problem is highlighted by the capacity of mammalian prion protein (PrP), a widely expressed and conserved GPI-anchored protein, to cause neurodegenerative disease when misfolded variants accumulate in cells (Prusiner, 2013).

The pathway used for degradation of a misfolded GPI-anchored protein depends on the step at which its biosynthesis fails. Errors in targeting GPI-anchored proteins to the endoplasmic reticulum (ER) or processing their signal for GPI anchor attachment at the membrane are handled by cytosolic (Ast et al., 2014; Hessa et al., 2011) and ER-associated degradation (ERAD) pathways (Ali et al., 2000; Ashok and Hegde, 2008; Sikorska et al., 2016; Wilbourn et al., 1998), respectively. Early studies in yeast suggested that once the GPI anchor is added, the misfolded protein is not degraded via Hrd1p (Fujita et al., 2006), a central ERAD factor that mediates ubiquitination and retrotranslocation of substrates from the ER to the cytosol (Baldridge and Rapoport, 2016; Bays et al., 2001; Stein et al., 2014).

Instead, misfolded GPI-anchored proteins in yeast were suggested to use a seemingly unconventional pathway dependent on ER-to-Golgi transport (Fujita et al., 2006). Subsequent analysis suggested that ER export receptors of the TMED family (also known as the p24 family) rapidly sequester GPI-anchored proteins to prevent their engagement by Hrd1p, thereby allowing degradation in the vacuole (Sikorska et al., 2016). The GPI-anchored protein was primarily degraded by ERAD when the TMED cargo receptor was eliminated. Thus, the primary pathway for GPI-anchored protein degradation in yeast is via trafficking to the vacuole, with ERAD serving as a failsafe pathway when the vacuole route is impaired.

Parallel studies in mammalian cells investigating mutant PrP degradation arrived at similar conclusions. First, investigation of the localization, trafficking, and turnover of a panel of human disease-causing PrP mutants showed that they are not degraded by ERAD, do not depend on the proteasome, and exit the ER despite their misfolding (Ashok and Hegde, 2009). The misfolded population of mutant PrP was selectively observed in post-ER intracellular compartments *en route* to their ultimate degradation in acidic compartments presumed to be lysosomes. Using an artificial constitutively misfolded PrP mutant (termed PrP*, containing a C179A mutation that cannot form the sole disulfide bond in PrP), trafficking from the ER to lysosomes was directly visualized by time-lapse imaging in live cells (Satpute-Krishnan et al., 2014). This study showed that PrP* is primarily retained in the ER at steady state but can be released into the secretory pathway by acute ER stress.

The steps between ER retention and lysosomal clearance are only partially understood. Transit of PrP* to the Golgi requires cargo receptor TMED10 (also known as Tmp21, or p24δ1) with which it interacts in co-immunoprecipitation experiments (Satpute-Krishnan et al., 2014). From here, the route to lysosomes is not established. At least a subpopulation was implicated in transiting the cell surface based on extracellular antibody uptake assays and trapping of PrP* at the cell surface after cholesterol depletion (Satpute-Krishnan et al., 2014). The proportion of PrP* using this itinerary was unclear but it is important to understand because exposing misfolded proteins to the extracellular environment can be detrimental. In the specific case of PrP, surface-exposed misfolded forms may facilitate uptake of prions into cells (Fehlinger et al., 2017).

From these combined studies in yeast and mammalian cells, it is thought that both folded and misfolded GPI-anchored proteins engage TMED family export receptors at the ER and traffic to the Golgi. At some step at or after the trans-Golgi network, their itineraries diverge. Folded GPI-anchored proteins go on to reside at the cell surface, whereas misfolded variants are delivered to the lysosome. It is not known how misfolded GPI-anchored proteins get from the Golgi to lysosomes, how they avoid aggregation during their journey through chaperone-poor post-ER compartments, or how cells discriminate folded from misfolded proteins to influence their trafficking. Here, we used quantitative flow cytometry and proteomic analyses to show that the majority of PrP* traffics via the cell surface to lysosomes in a complex with resident ER chaperones and cargo receptors. This suggests that minor populations of abundant factors long thought to be restricted to the early secretory pathway have functional excursions to the cell surface during quality control of GPI-anchored proteins.

## Results

### Experimental system for quantitative analysis of PrP* degradation

**Figure 1.**
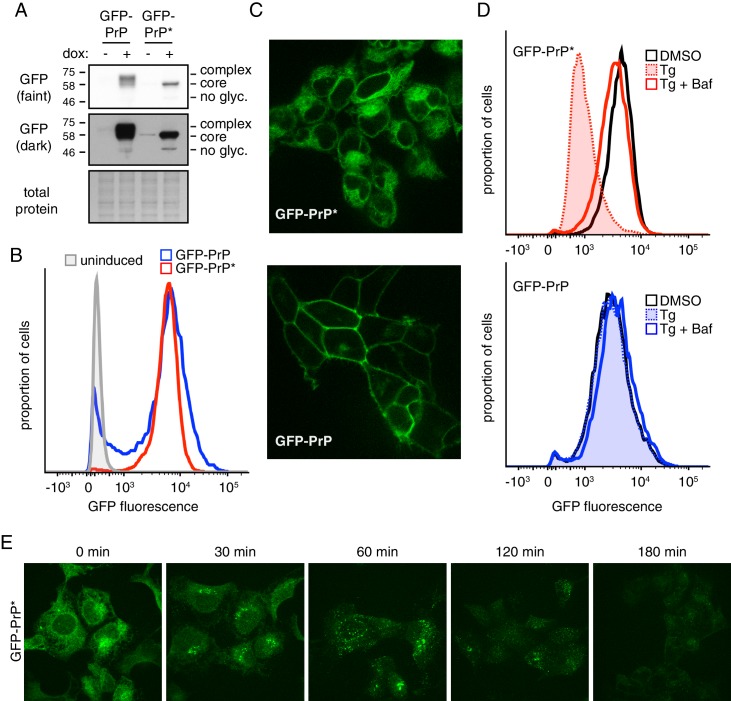
A stable-inducible cell line to study GPI-anchored protein quality control. (**A**) HEK293-TRex cells containing either GFP-PrP or GFP-PrP* stably integrated at the same locus were induced to express the proteins with doxycycline for 48 hr prior to analysis by immunoblotting using anti-GFP antibody. Cultures without doxycycline induction were analyzed in parallel. Two exposures of the immunoblot are shown, along with a portion of the stained blot verifying equal loading. Unglycosylated (‘no glyc.”), core-glycosylated (‘core’), and complex-glycosylated (‘complex’) species of GFP-PrP are indicated. (**B**) Cells expressing GFP-PrP or GFP-PrP* were induced with doxycycline for 96 hr prior to analysis of GFP fluorescence by flow cytometry. The normalized histograms are shown. (**C**) Cells expressing GFP-PrP or GFP-PrP* were induced for 48 hr with doxycycline, washed to remove doxycycline, then analyzed 24 hr later by fluorescent microscopy. Doxycycline withdrawal for 24 hr limited the amount of reporter mRNA in cells to relatively moderate levels, and was employed in most of the experiments in this study. This minimized over-expression artifacts and improved reproducibility. (**D**) Cells expressing GFP-PrP or GFP-PrP* were treated with vehicle (DMSO) or the ER stressor thapsigargin (Tg, at 100 nM) for 3 hr in the presence of 100 µg/ml cycloheximide to suppress new protein synthesis. 250 nM bafilomycin A1 (BafA1) was included in one set of samples. Total GFP fluorescence was measured by flow cytometry. (**E**) Cells expressing GFP-PrP* were treated with 100 nM thapsigargin (Tg) and 100 µg/ml cycloheximide and imaged at the indicated times.

To perform quantitative analysis of misfolded GPI-anchored protein degradation, we first generated and characterized a stable doxycycline-inducible HEK293T cell line expressing GFP-tagged PrP* (GFP-PrP*) integrated into a single defined locus in the genome. This mutant of PrP contains a Cys to Ala change at position 179, thereby preventing the formation of a critical disulfide bond required for PrP folding (Satpute-Krishnan et al., 2014). A matched cell line expressing wild type GFP-PrP from the same locus served as a control in these studies. Immunoblotting of total cell lysates after induction with doxycycline showed that the steady state level of GFP-PrP* was very similar to GFP-PrP (Figure 1A). The different migration patterns are due to complex glycosylation of GFP-PrP during its transit through the Golgi in contrast to core-glycosylated GFP-PrP* primarily retained in the ER.

**Figure 1—figure supplement 1.**
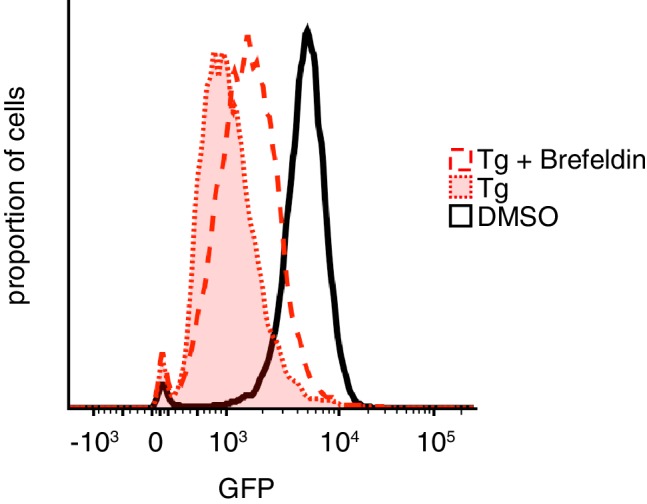
Effect of Brefeldin A on GFP-PrP* degradation. Cells expressing GFP-PrP* were treated for 3 hr with vehicle (DMSO) or 100 nM thapsigargin (Tg) without or with 2.5 µg/ml Brefeldin A. 100 µg/ml cycloheximide was included in all samples during the 3 hr treatment to suppress new protein synthesis. Total GFP fluorescence was measured by flow cytometry.

Consistent with the immunoblotting, flow cytometry of GFP fluorescence showed that total GFP-PrP* was almost identical to GFP-PrP (Figure 1B). As expected from earlier studies (Satpute-Krishnan et al., 2014), GFP-PrP* was primarily localized intracellularly in a pattern consistent with the ER, while GFP-PrP was primarily at the cell surface (Figure 1C). Upon induction of acute ER stress with thapsigargin (Tg),~80% of GFP-PrP* was degraded after three hours (Figure 1D, upper panel). This degradation was almost completely inhibited by Bafilomycin A1 (BafA1), which prevents lysosome acidification and reduces proteolytic activity. By contrast, GFP-PrP was not affected by Tg and was only slightly stabilized by BafA1 (Figure 1D, lower panel) consistent with its slow turnover from the cell surface in lysosomes.

Microscopy of GFP-PrP* cells at different times after ER stress showed re-localization from the ER to a peri-nuclear structure to cytosolic puncta before degradation (Figure 1E). Based on earlier work (Satpute-Krishnan et al., 2014), this corresponds to GFP-PrP* trafficking via the Golgi to lysosomes. While Brefeldin A (BFA) causes ER retention of GFP-PrP* and precludes trafficking to lysosomes (as verified by microscopy; data not shown), degradation still proceeds, albeit somewhat less efficiently (Figure 1—figure supplement 1). Based on earlier studies in yeast (Sikorska et al., 2016), it is likely that in the absence of ER export, GFP-PrP* is eventually degraded by ERAD. Thus, our matched stable-inducible cell lines recapitulate the previously described stress-triggered PrP* degradation in lysosomes and the comparatively stable cell surface residence of PrP. These tools provided the foundation for quantitative analyses of PrP* quality control, trafficking, and degradation.

### Detection and quantification of PrP* at the cell surface

**Figure 2.**
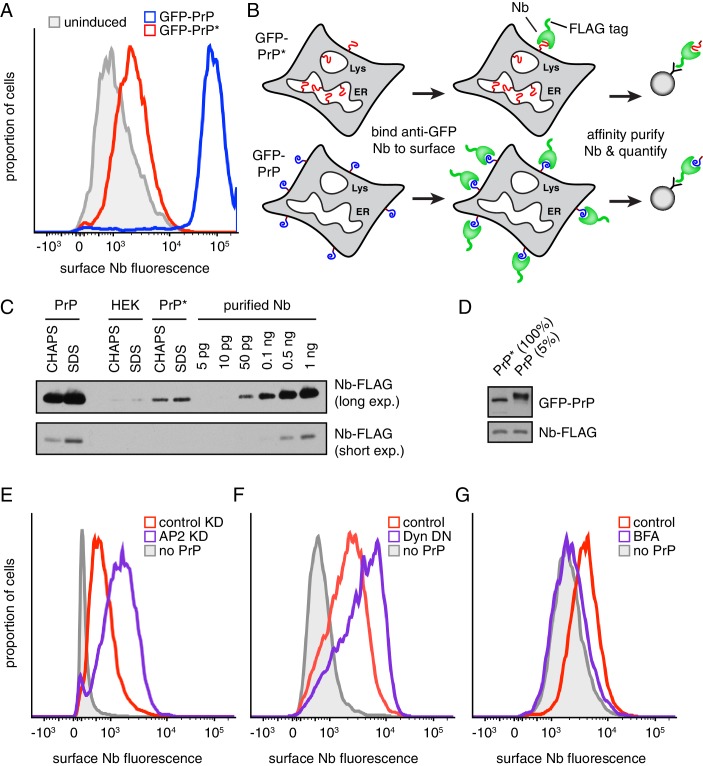
Misfolded PrP is detectable at the cell surface prior to lysosomal degradation. (**A**) Cells expressing GFP-PrP and GFP-PrP* were incubated on ice with 200 nM extracellular Alexa647-conjugated Nb and Alexa647 fluorescence was measured by flow cytometry. Uninduced cells were analyzed in parallel and are shown in gray. (**B**) Diagram of experimental strategy to compare relative surface levels of GFP-PrP* and GFP-PrP. (**C**) Cells expressing GFP-PrP and GFP-PrP* were labeled with saturating amounts of anti-GFP Nb on ice, washed, and lysed under denaturing (SDS) or non-denaturing (CHAPS) conditions. Bound Nb was analyzed by immunoblotting relative to serial dilutions of purified recombinant Nb. (**D**) The indicated relative amounts of surface-derived complexes purified under non-denaturing conditions were analyzed by immunoblotting for GFP and FLAG. (**E**) GFP-PrP*-expressing cells treated for 96 hr with control or AP2-targeting siRNAs were surface-labeled with Alexa647-Nb and analyzed by flow cytometry. HEK293T cells without GFP-PrP* were analyzed for comparison (gray). (**F**) GFP-PrP*-expressing cells were transiently transfected with FusionRed-Dynamin S45N (Almeida-Souza et al., 2018) or empty vector for 24 hr prior to surface staining with Alexa647-Nb and analysis by flow cytometry. (**G**) GFP-PrP*-expressing cells were treated with 2.5 µg/ml Brefeldin A (BFA) for two hours prior to surface staining with Alexa647-Nb and analysis by flow cytometry.

To monitor GFP-PrP at the cell surface, we prepared purified recombinant anti-GFP camelid-derived nanobody (Nb) labeled with a chemical fluorophore and 3X-FLAG epitope as an affinity handle. The Nb-FLAG probe is small (~18 kD), monovalent, exceptionally specific, has sub-nM affinity, very fast on-rate, and very slow off-rate. These properties make it an ideal probe for detecting but not crosslinking surface-exposed GFP. Titration experiments showed that extracellular Nb concentrations above 10 nM saturate surface labeling of GFP-PrP (Figure 2—figure supplement 1A). Using saturating concentrations of extracellular Nb, we found that cells expressing GFP-PrP* contain a clearly detectable surface population whose level is between one-twentieth and one-fiftieth of cells expressing GFP-PrP (Figure 2A).

**Figure 2—figure supplement 1.**
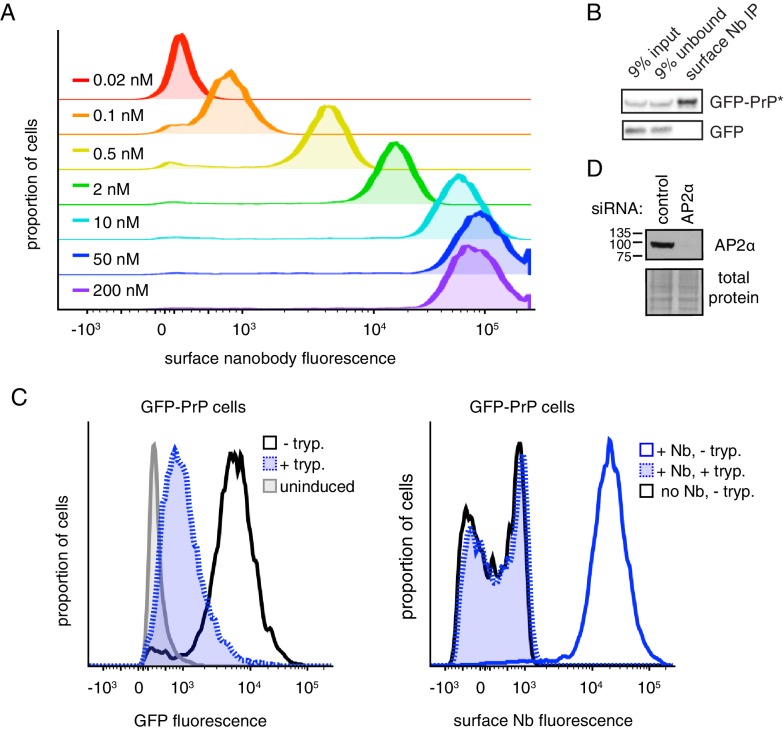
Characterization of Nb surface staining and isolation. (**A**) Cells expressing GFP-PrP were incubated on ice with varying concentrations of Alexa647-Nb, washed, and analyzed for Alexa647 fluorescence by flow cytometry. (**B**) GFP-PrP* on the cell surface was labeled on ice with Nb-FLAG, washed, and lysed under non-denaturing conditions. The lysate was then mixed with lysate from cells transfected with cytosolic GFP. The nanobody was subsequently immunoprecipitated (IP) via its FLAG epitope tag. The input, unbound and IP samples were analyzed by SDS-PAGE and immunoblotting using anti-GFP antibodies. No cytosolic GFP was recovered in the IP, illustrating that the Nb bound to surface GFP-PrP* does not exchange antigens after solubilization and during the IP. (**C**) GFP-PrP-expressing cells were incubated without or with extracellular trypsin to digest surface-localized proteins. The left panel shows the level of total GFP fluorescence analyzed by flow cytometry relative to uninduced cells. In the right panel, the trypsin-digested or undigested samples were surface labeled with fluorescent Nb and analyzed by flow cytometry. Note that surface GFP-PrP was quantitatively digested by trypsin as indicated by the fact that these cells show no detectable Nb fluorescence relative to cells that were not labeled with Nb. Thus, we can deduce that the remaining GFP fluorescence in the trypsin-digested cells seen in the left panel is due to the intracellular population of GFP-PrP. (**D**) Verification of AP2 knockdown after siRNA treatment, as part of the experiment in Figure 2E.

To quantify this minor surface population, we compared the amount of surface-bound Nb to serial dilutions of purified Nb (Figure 2B and C). As expected, cells that did not express GFP-PrP* or GFP-PrP did not yield appreciable Nb. Cells expressing GFP-PrP* yielded one-twentieth the amount of Nb relative to cells expressing GFP-PrP (50 pg and 1 ng, respectively). When the surface-bound Nb was purified under non-denaturing conditions, GFP-PrP* was co-precipitated as expected (Figure 2D). Consistent with the above quantification, analysis of Nb-bound GFP-PrP* relative to 5% of Nb-bound GFP-PrP showed equal amounts of GFP signal by immunoblotting (Figure 2D). Importantly, control experiments in which lysate from Nb-labeled cells was mixed with lysates containing free GFP showed that free GFP was not recovered in the Nb-purified complexes (Figure 2—figure supplement 1B). This verified that surface-bound Nb does not exchange antigens after cell lysis over the time frame needed for affinity purification of Nb-antigen complexes. Thus, 20-fold more PrP resides at the cell surface than PrP*.

Based on sensitivity to extracellular trypsin, we could determine that 87% of total GFP-PrP is at the cell surface (Figure 2—figure supplement 1C). Given that total GFP-PrP* is equal to total GFP-PrP (Figure 1A–1C), we can deduce that ~4.4% of GFP-PrP* is at the cell surface (i.e., one-twentieth of 87%). Most of the remaining 96% of GFP-PrP* is located in the ER (Figure 1C). Despite this very low steady state level at the surface, two additional experiments validated its existence. First, the surface level increased when endocytosis was impaired by either knocking down AP2 (Figure 2E, Figure 2—figure supplement 1D) or expressing a dominant-negative Dynamin (Damke et al., 2001) (Figure 2F). Second, the surface population was decreased to near-background levels by blocking ER exit with BFA for two hours (Figure 2G). Thus, by both flow cytometry and direct affinity purification,~4.4% of GFP-PrP* is at the cell surface at steady state. This population is apparently transient since it is lost shortly after further ER-to-surface trafficking is blocked (Figure 2G). Curiously, almost all of the surface population of GFP-PrP* contains immature glycans (Figure 2D) despite having trafficked through the Golgi (see Discussion).

### Nearly all PrP* transits the cell surface during its degradation

**Figure 3.**
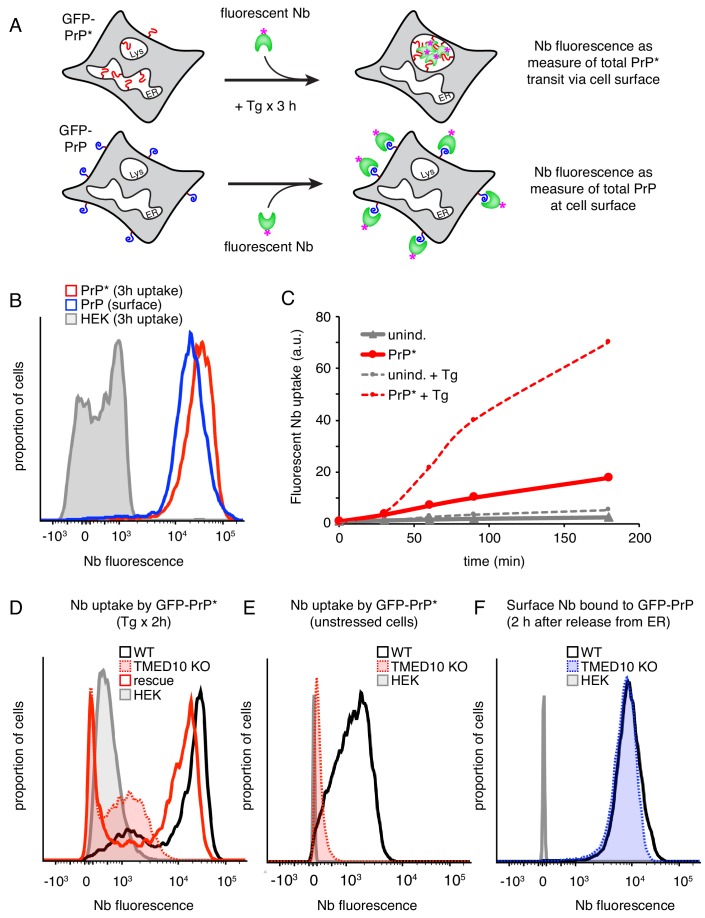
The majority of misfolded PrP transits the cell surface en route to lysosomes. (**A**) Experimental strategy to measure GFP-PrP* transit through the cell surface (top) relative to a known standard generated by saturating surface labeling of GFP-PrP cells. (**B**) Cells expressing GFP-PrP* were incubated for 3 hr at 37°C with 100 nM Tg and 10 nM extracellular Alexa546-Nb prior to washing and analysis by flow cytometry. This sample was compared to GFP-PrP cells surface-labeled with Alexa546-Nb. (**C**) GFP-PrP*-expressing cells and uninduced controls were incubated for various times in the presence of 10 nM Alexa546-Nb in the culture medium. Half the cells contained 100 nM thapsigargin (Tg) while the other half were untreated. The cells at each time point were analyzed by flow cytometry. The graph depicts the median fluorescence intensity of internalized nanobody relative to time 0. (**D**) Wild type (WT) and TMED10 knockout (KO) cells expressing GFP-PrP* were allowed to internalize Alexa647-Nb from the extracellular medium for 2 hr during treatment with 100 nM thapsigargin. Fluorescent Nb levels were measured by flow cytometry. KO cells transiently transfected with HA-tagged TMED10 (rescue) were analyzed in parallel. (**E**) The amount of extracellular Nb uptake was measured over the course of 2 hr in unstressed cells expressing GFP-PrP*. Wild type (WT) cells were compared to TMED10 knockout cells (KO). Cells lacking GFP-PrP* (HEK) were analyzed as a negative control. (**F**) GFP-PrP-expressing cells were treated with 1 µM Brefeldin A (BFA) for 2.5 hr to retain GFP-PrP in the ER. The media was then changed to remove BFA while adding Alexa647-Nb, and incubated for 2 hr before analysis by flow cytometry.

The ability to increase and decrease the surface population of GFP-PrP* by manipulating trafficking pathways provides qualitative evidence that it is constantly delivered to and degraded from the plasma membrane. However, the numerous pleiotropic consequences of inhibiting major trafficking pathways preclude this strategy for deducing the proportion of total synthesized GFP-PrP* accessing the surface. We therefore sought to estimate GFP-PrP* flux through the cell surface in trafficking-competent cells. The strategy we used was to directly measure the total amount of extracellular Nb taken up by cells during a time period when the entire ER pool of GFP-PrP* traffics to lysosomes (Figure 3A, top diagram). This value was compared to a fluorescence standard generated in parallel by surface-stained GFP-PrP cells using the same Nb (Figure 3A, bottom diagram).

**Figure 3—figure supplement 1.**
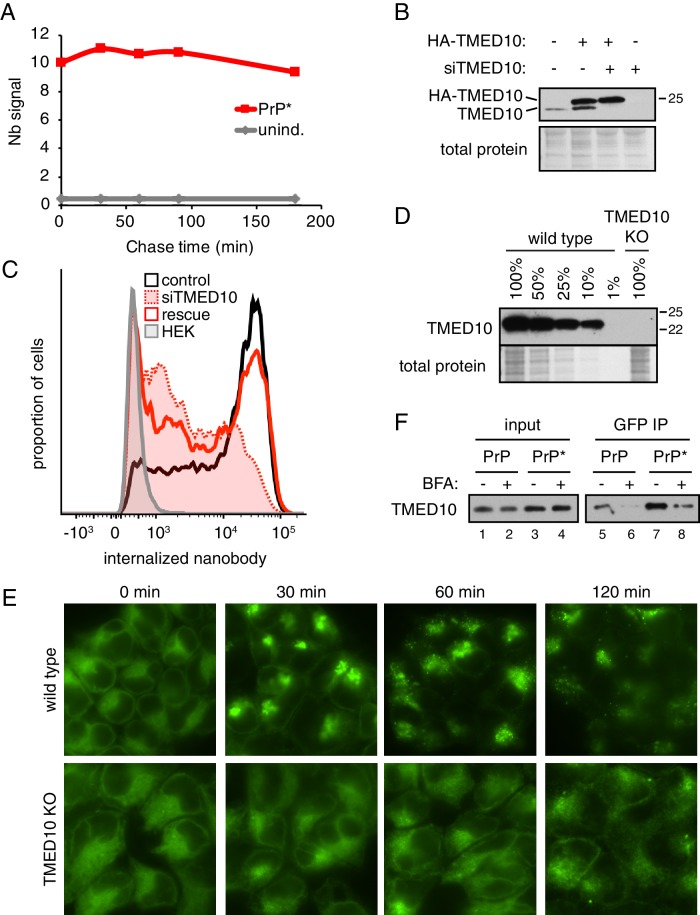
Additional characterization of GFP-PrP* trafficking. (**A**) Cells expressing GFP-PrP* (or uninduced controls) were allowed to internalize Alexa546-Nb for 3 hr, after which the cells were washed, put into Nb-free medium, and further cultured for the indicated times. Nb fluorescence was measured by flow cytometry at each time point and the median fluorescence intensity is plotted. Note that no appreciable Nb uptake is seen in uninduced cells expressing minimal amounts of GFP-PrP*, and that the fluorescence of internalized Nb does not change appreciably over the course of 3 hr. (**B**) GFP-PrP*-expressing cells were treated without or with siRNAs targeting TMED10 (siTMED10), and transfected with a control vector or one expressing siRNA-resistant HA-TMED10. After allowing 96 hr to fully deplete endogenous TMED10, the cells were harvested and analyzed by immunoblotting for TMED10, which reveals both endogenous and exogenous TMED10. (**C**) GFP-PrP*-expressing cells were treated without (control) or with siRNAs targeting TMED10 (siTMED10) for 96 hr as in panel B. A subset of knockdown cells was also transfected with siRNA-resistant HA-TMED10 (rescue). Each of these cells, along with untreated HEK293T cells (HEK) were treated for 2 hr with 100 nM thapsigargin in the presence of extracellular Alexa546-Nb. Fluorescent nanobody uptake was measured by flow cytometry. (**D**) The levels of TMED10 in unedited (wild type) and CRISPR-edited TMED10-knockout (TMED10 KO) cells was assessed by immunoblotting (using an antibody against the lumenal domain). The KO lysate was compared to serial dilutions of control lysate to verify that no TMED10 was detectable in the KO cells even with over-exposure of the blot. (**E**) WT and ∆TMED10 cells (see panel D) expressing GFP-PrP* were imaged by fluorescence microscopy at the indicated times after treatment with 100 nM thapsigargin to induce acute ER stress. Unlike in wild type cells, GFP-PrP* in ∆TMED10 cells remains in the ER over this time frame. (**F**) Cells expressing GFP-PrP and GFP-PrP* were lysed under non-denaturing conditions and immunoprecipitated using sepharose-conjugated anti-GFP Nb. Input lysates and elutions were immunoblotted for TMED10.

Based on direct imaging (Figure 1E) and the time point when most PrP* is degraded in lysosomes (Figure 1D), we decided to monitor Nb uptake during an acute 3 hr ER stress treatment of GFP-PrP* cells. Due to a combination of transcriptional shutoff prior to the experiment and translation attenuation during acute ER stress, new GFP-PrP* synthesis during the time course is relatively minor compared to the pre-existing population. Furthermore, we verified in preliminary experiments that uptake in the absence of GFP-PrP* expression is negligible over 3 hr without or with ER stress (data not shown; see also Figure 3B, gray trace). Finally, we confirmed that the fluorophore on the Nb remains quantitatively fluorescent and intracellular for at least 3 hr after uptake even if GFP-PrP* and the Nb are being degraded in lysosomes (Figure 3—figure supplement 1A). These considerations allow us to assign essentially all nanobody uptake during the time course to GFP-PrP* having accessed extracellular anti-GFP nanobody.

GFP-PrP* cells at steady state, as characterized in Figure 1, were treated for 3 hr with acute ER stress (0.1 µM thapsigargin) in the presence of extracellular fluorescent Nb. The cells were then washed and the total amount of nanobody uptake was quantified by flow cytometry relative to the Nb-labeled GFP-PrP fluorescence standard. We observed that the nanobody taken into GFP-PrP* cells (Figure 3B, red trace) slightly exceeded the GFP-PrP fluorescence standard (Figure 3B, blue trace). The fluorescence standard represents the quantity of nanobody binding that corresponds to 87% of total GFP-PrP. At the start of the experiment, the amount of GFP-PrP* available for nanobody binding is ~4.4% (i.e., 20-fold less than our fluorescence standard). Thus, for GFP-PrP* cells to accumulate an amount of nanobody comparable to the fluorescence standard,~86% of the intracellular population would need to access and trap extracellular nanobody. Given that Nb accumulation in GFP-PrP* cells actually exceeds the GFP-PrP standard (Figure 3B), we conclude that the vast majority of initially intracellular GFP-PrP* must access the cell surface on its way to lysosomes during stress-triggered degradation. As expected for a trafficking itinerary via the Golgi, Nb uptake displayed a lag of ~30 min consistent with the transit time for GFP-PrP* from the ER to the cell surface (Figure 3C).

In the absence of ER stress, GFP-PrP* cells also take up extracellular nanobody selectively when GFP-PrP* is expressed (Figure 3C). This suggests that even at steady state, GFP-PrP* accesses the cell surface similarly to what is observed during stress. The slopes of the uptake time courses between 30 and 90 min suggest that the rate of surface access is ~6 fold higher during acute ER stress than at steady state. The basis of this difference is likely due to the different rates of ER egress under unstressed and stressed conditions. This result suggested the possibility that GFP-PrP* degradation during acute stress is an accelerated version of the degradation pathway under normal conditions.

To investigate this idea, we tested whether TMED10, previously implicated in stress-triggered PrP* export from the ER (Satpute-Krishnan et al., 2014; Sikorska et al., 2016), is similarly required under unstressed conditions. In preliminary studies, we found that acute knockdown of TMED10 (Figure 3—figure supplement 1B) impaired uptake of extracellular Nb (Figure 3—figure supplement 1C), an indicator of reduced GFP-PrP* flux through the cell surface. Gene editing was then used to knock out TMED10 in GFP-PrP* cells (Figure 3—figure supplement 1D). Fluorescence microscopy showed that in ∆TMED10 cells, acute ER stress no longer caused GFP-PrP* egress from the ER (Figure 3—figure supplement 1E) and sharply impaired uptake of extracellular Nb (Figure 3D). Using the uptake assay, we found that Nb accumulation was also impaired under non-stressed conditions, further suggesting that the same pathway is used, albeit at a lower rate (Figure 3E). Importantly, little or no effect was observed on the amount of GFP-PrP trafficked to the cell surface in ∆TMED10 cells (Figure 3F) indicating that neither trafficking nor GPI-anchored protein biogenesis are appreciably impaired. Thus, although both PrP* and PrP interact with TMED10 by coimmunoprecipitation (Figure 3—figure supplement 1F), only PrP* strongly relies on it for lysosomal degradation via the cell surface.

### A complex of p24 proteins is required for PrP* trafficking and degradation

**Figure 4.**
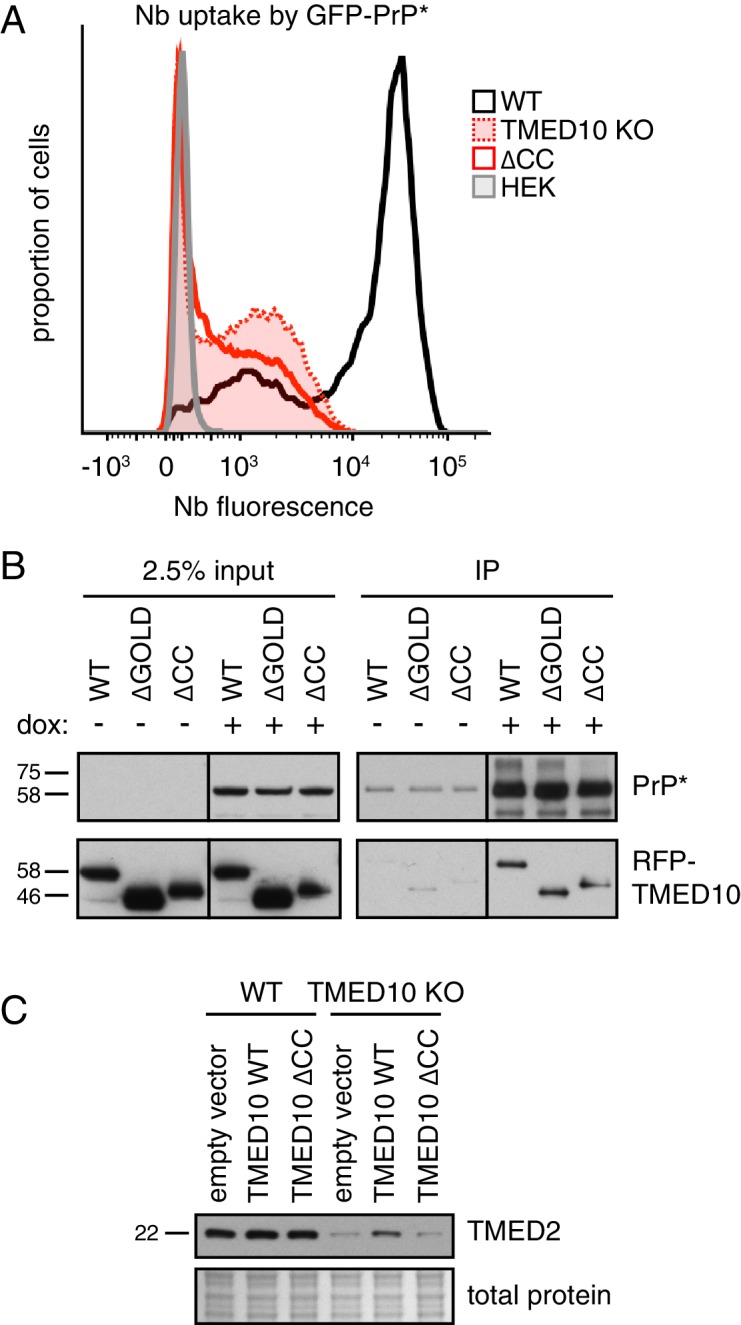
A complex of several TMED proteins facilitates GFP-PrP* degradation. (**A**) Wild type (WT) or TMED10 knockout (KO) cells expressing GFP-PrP* were assayed for thapsigargin-stimulated extracellular Nb uptake as in Figure 3D. In addition, the KO cells were transiently transfected with HA-tagged TMED10 lacking its coiled-coil domain (∆CC) and the transfected cells were analyzed in parallel. (**B**) RFP-tagged wild type (WT) TMED10, or constructs lacking the GOLD domain (residues 41–129; ∆GOLD) or coiled-coil domain (residues 130–183; ∆CC) were transiently transfected into ∆TMED10 cells inducibly expressing GFP-PrP*. GFP-PrP* was either left uninduced or induced for 48 hr prior to analysis. GFP-PrP* was immunoprecipitated using sepharose-conjugated anti-GFP Nb and analyzed by immunoblotting for GFP and RFP relative to input lysates. All input samples are from the same blot and exposure, with the vertical line indicating where intervening lanes were removed. All of the IP samples are also from the same blot and exposure. (**C**) Wild type or TMED10 knockout cells were transiently transfected with RFP-tagged WT or ∆CC TMED10. Two days post-transfection, cells containing moderate levels of RFP were isolated by flow cytometry, lysed, and immunoblotted against TMED2.

TMED10 sedimentation through a sucrose gradient suggests a native size larger than its 24 kD molecular weight (Figure 4—figure supplement 1A), consistent with it being part of a multi-protein complex (Jenne et al., 2002). Earlier work suggested that the coiled-coil region of the TMED family is important for homo- and hetero-typic interactions (Ciufo and Boyd, 2000; Emery et al., 2000). To test whether the role of TMED10 in GFP-PrP* degradation depends on complex formation, we assessed the ability of an assembly mutant lacking the coiled-coil (∆CC) to rescue the phenotype of ∆TMED10 cells. While ∆TMED10 cells rescued with wild type TMED10 largely recovered Nb uptake by GFP-PrP* (Figure 3D and Figure 4—figure supplement 1B), ∆CC was completely inactive (Figure 4A) despite unimpaired interaction with GFP-PrP* (Figure 4B). Of note, deletion of the lumenal GOLD domain implicated in protein-protein interactions of uncertain relevance was functional in restoring GFP-PrP* trafficking to ∆TMED10 cells (data not shown) and unimpaired in its interaction with GFP-PrP* (Figure 4B). These observations can be explained if GFP-PrP* trafficking relies on a complex of multiple homologous TMED family proteins, more than one of which can interact with GFP-PrP*. The precise identity of the cargo receptor complex employed by GFP-PrP* and the nature of its selective engagement of misfolded GPI-anchored proteins remains to be defined.

**Figure 4—figure supplement 1.**
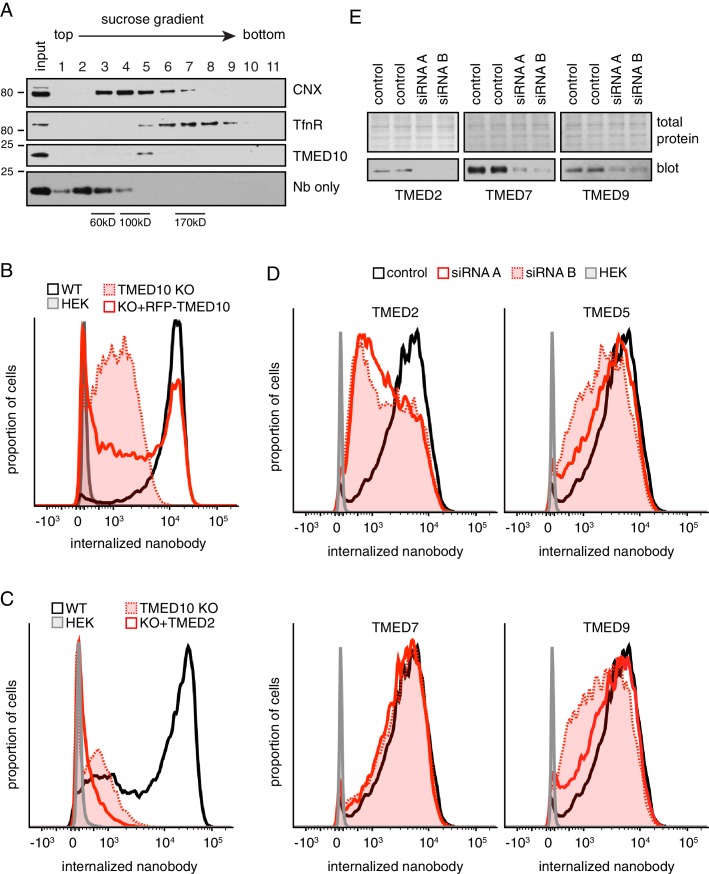
Additional characterization of TMED family members. (**A**) Cell lysates prepared under non-denaturing conditions were separated by size on a 5–25% sucrose gradient, and analyzed by immunoblotting. Purified recombinant nanobody (18 kDa) peaks in fraction 2, as expected for a small monomeric protein. Despite a similar molecular weight, native TMED10 peaks in fraction five indicating its constitutive engagement in a higher molecular weight complex. For comparison, CNX (~90 kDa monomer) peaks in fraction 4, while the transferrin receptor (TfnR, dimer of two 85 kDa polypeptides) peaks in fractions 6–8. This suggests that native TMED10 is part of a ~ 100–125 kD complex. (**B**) Wild type (WT) cells, TMED10 knockout (KO) cells, KO cells transiently transfected with RFP-tagged TMED10, and HEK293 cells were assayed for their ability to internalize fluorescent nanobody for 1 hr in the presence of thapsigargin-induced ER stress. Nanobody fluorescence was measured by flow cytometry. RFP-tagged TMED10 is able to partially rescue nanobody internalization in ∆TMED10 cells. (**C**) GFP-PrP*-expressing cells lacking endogenous TMED10 (KO) were transiently transfected with myc-tagged TMED2 and assayed for their ability to internalize fluorescent nanobody for 2 hr in the presence of thapsigargin-induced ER stress. TMED2 cannot rescue the Nb-uptake deficiency of ∆TMED10 cells, and seems to even have a slight dominant-negative effect (i.e., further reduced Nb uptake). (**D**) Cells expressing GFP-PrP* were depleted of the indicated TMED family members using two independent siRNA oligonucleotides for 72 hr, and subsequently assayed for their ability to internalize fluorescent nanobody from the medium over a 100 min time period under non-stressed conditions. Although not shown here, no effect was seen for siRNA-treated TMED1, TMED3, TMED4, and TMED6. (**E**) Knockdown efficiencies for panel D were checked by immunoblotting for TMED2, TMED7, and TMED9. We were unable to obtain a suitable antibody for TMED5.

Consistent with TMED10 interacting with other TMED family member(s), ∆TMED10 cells had reduced levels of TMED2, a putative part of the complex (Figure 4C). Reduced TMED2 is partially restored upon acute re-introduction of TMED10 by transient transfection, but not by re-introduction of the ∆CC mutant defective in hetero-typic TMED family interactions. Notably, the phenotype of ∆TMED10 cells cannot be rescued by TMED2 over-expression arguing against TMED2 loss as the sole basis of impaired GFP-PrP* trafficking (Figure 4—figure supplement 1C). Analysis of Nb uptake by GFP-PrP* in cells treated with siRNAs against each TMED family member suggests that in addition to TMED10, TMED2 is strongly required (Figure 4—figure supplement 1D,E). A weaker phenotype is observed with at least one siRNA for both TMED5 and TMED9, but not TMED7. The combination of TMED2/5/9/10 was previously reported to participate in the ER exit of GPI-anchored proteins (Fujita et al., 2011). Our findings suggest that GFP-PrP* trafficking similarly requires TMED2 and TMED10, possibly in a complex that also includes TMED5 and TMED9. A systematic analysis of the complete cargo receptor complex for misfolded GPI-anchored proteins requires further study.

### The PrP* interactome during ER-to-lysosome trafficking

The data thus far have established that GFP-PrP* constitutively (but slowly) leaves the ER in a TMED10-dependent manner and traffics via the cell surface to lysosomes. By contrast, GFP-PrP efficiently folds, leaves the ER, and resides primarily at the plasma membrane from where it slowly turns over in lysosomes. To understand the basis of these different itineraries, we systematically analyzed PrP*-specific interactions in both the ER and post-ER compartments.

**Figure 5.**
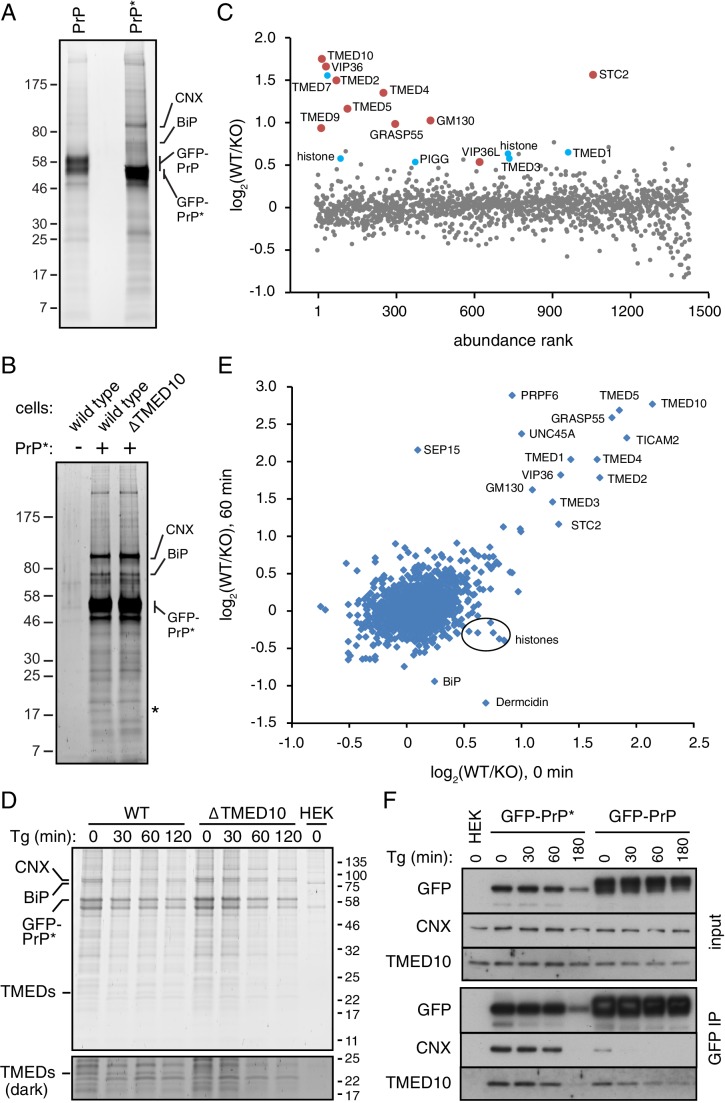
Proteomic analysis of PrP* interactions during its trafficking to lysosomes. (**A**) Cells expressing GFP-PrP and GFP-PrP* were lysed under non-denaturing conditions, affinity purified via immobilized anti-GFP Nb, separated by SDS-PAGE, and stained with SYPRO-Ruby. The positions of key proteins (verified by mass spectrometry) are shown. (**B**) GFP-PrP* from wild type and ∆TMED10 cells was affinity purified under non-denaturing conditions, separated by SDS-PAGE, and stained with SYPRO-Ruby. The asterisk indicates the only band obviously absent in the ∆TMED10 sample, presumably corresponding to TMED10 itself. (**C**) GFP-PrP* was immunoprecipitated from wild type (WT) and ∆TMED10 (KO) cells as in panel B and analyzed by quantitative mass spectrometry. The x-axis orders proteins by relative abundance in the immunoprecipitation (one being the most abundant), and the y-axis plots the Log_2_ value of the WT/KO ratio of abundances. Proteins whose Log_2_ ratios are >0.5 in all three independent experiments are highlighted in red, and those >0.5 in 2 of 3 independent experiments are highlighted in blue. (**D**) GFP-PrP* was immunoprecipitated as in panel B from wild type (WT) and ∆TMED10 (KO) cells at different times after thapsigargin-induced acute ER stress. HEK293T cells lacking GFP-PrP* were used as a specificity control (HEK). The positions of several abundant proteins identified by mass spectrometry are indicated. The portion of the gel showing the TMED family members is also shown at higher contrast. All eight IP samples (not including the HEK control) were subjected to quantitative tandem mass tagging (TMT) mass spectrometry (Figure 5—source data 1). (**E**) The quantitative mass spectrometry results from the ‘0’ time point and the ‘60 min’ time point from the experiment in panel D are plotted as Log_2_ values of the WT/KO ratio. Various proteins discussed in the text are indicated. (**F**) GFP-PrP and GFP-PrP* were immunoprecipitated under non-denaturing conditions following thapsigargin-induced ER stress for the indicated time periods. Input and immunoprecipitated samples were separated by SDS-PAGE and analyzed by immunoblotting against CNX, TMED10, and GFP. 10.7554/eLife.46740.012Figure 5—source data 1.Complete tandem mass tagging mass spectrometry data.The eight samples corresponding to the four time points each for WT and ∆TMED10 cells from Figure 5D were analyzed by tandem mass tagging (TMT) quantitative proteomics and the data tabulated in the Excel table. Each tab of the Excel table illustrates the sequential steps in the normalization and analysis of the raw data, ending in the graph depicted in Figure 5E.

Using the GFP tag as an affinity handle, we first purified GFP-PrP and GFP-PrP* from non-stressed cells at steady state and compared their interaction partners. As observed in two examples (Figure 5A and Figure 5—figure supplement 1A), GFP-PrP* specifically co-precipitated a variety of proteins that were reduced or absent from cells expressing GFP-PrP. As expected for a misfolding mutant, mass spectrometry identified a number of ER-resident chaperones that were co-precipitated preferentially with GFP-PrP*. The most abundant of these were Calnexin (CNX) and BiP (also called GRP78), with lesser amounts of GRP94 (also called endoplasmin), protein disulphide isomerase (PDI), ERP57 (an oxidoredutase that cooperates with CNX), and ERP72. A wide range of other interaction partners were recovered with GFP-PrP* at lower levels, but their specificity and relevance were less obvious.

**Figure 5—figure supplement 1.**
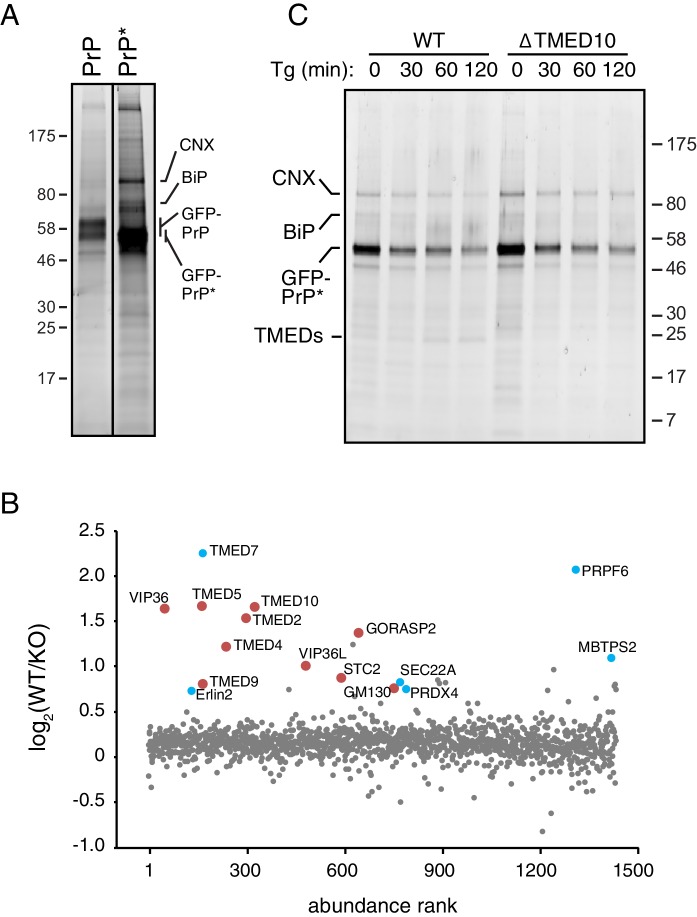
Additional analysis of GFP-PrP* interaction partners. (**A**) Independent replicate of the affinity purification experiment shown in Figure 5A. The two lanes corresponding to GFP-PrP and GFP-PrP* are from the same gel and exposure, with the vertical line indicating where intervening lanes removed. (**B**) Independent replicate of the GFP-PrP* interactome in WT and ∆TMED10 cells as shown in Figure 5C. (**C**) Independent replicate of the time course of GFP-PrP* interactions in WT and ∆TMED10 cells shown in Figure 5D.

To determine which (if any) of these other potential interacting partners might engage PrP* after it leaves the ER, we performed a quantitative mass spectrometry analysis of PrP*-interacting proteins from wild type and ∆TMED10 cells. We reasoned that the impaired ER exit of PrP* in ∆TMED10 cells would preclude biologically relevant post-ER interactions, whereas ER-resident interactors (or those recovered solely due to the ‘sticky’ nature of PrP*) would be unchanged. As expected, the stained gel shows that the major interactors (i.e., with resident ER chaperones) are essentially unchanged (Figure 5B), consistent with the principal location of PrP* in the ER in both cases. A conspicuous band between 17 kD and 25 kD, corresponding to the size of most TMED family members, was specifically absent in the ∆TMED10 sample.

Direct and quantitative comparison of the two samples by tandem-mass-tag (TMT) mass spectrometry from three independent biological replicates identified between ~1400 and~2000 proteins (Figure 5C, Figure 5—figure supplement 1B, and data not shown). For each of these, the relative amount recovered with GFP-PrP* from wild type (WT) cells versus ∆TMED10 cells (KO) was calculated (i.e., the WT/KO ratio, normalized so that the PrP ratio is 1, displayed as a Log_2_ value). Ten proteins showed a WT/KO ratio of least 1.4 (i.e., Log_2_ of 0.5) from all three TMT experiments, and another 11 proteins exceeded this threshold in two of three experiments (six were found in the experiment displayed in Figure 5C). Of these 21 proteins, seven were TMED family members involved in bidirectional trafficking between the ER and Golgi. Notably, the four family members implicated in functional assays of GFP-PrP* trafficking (TMED10, TMED2, TMED5, and TMED9) were among the proteins enriched in wild type cells in all three TMT experiments. This can be explained by lower TMED family abundance and/or reduced interaction with PrP* in ∆TMED10 cells.

Very few other post-ER components of the secretory or endocytic pathways showed a WT/KO ratio above 1.4. Two homologous lectins involved in ER-Golgi protein trafficking (VIP36 and VIP36L) and a putative secreted calcium binding protein (Stannocalcin2) of unclear function were the only proteins within the secretory pathway that could realistically encounter PrP* in post-ER compartments. Some components such as Sec22A, GM130 (also called GOLGA2), or GRASP55 (also called GORASP2) might interact indirectly via the TMED cargo receptors or the trafficking lectins, while the remaining proteins with elevated WT/KO ratios are likely to be artefacts. Notably, no cell surface or endosomal membrane proteins were found that could represent interacting partners during the post-Golgi steps of PrP*’s trafficking itinerary to lysosomes.

To specifically enrich for such post-ER interactors, we analyzed GFP-PrP* interactors using TMT mass spectrometry from wild type and ∆TMED10 cells at different times after acute ER stress. GFP-PrP* leaves the ER rapidly after stress, is preferentially enriched in Golgi and post-Golgi compartments at 30–60 min, and degraded beginning shortly after 60 min (Figure 1E). These post-ER locations are effectively inaccessible to GFP-PrP* in ∆TMED10 cells even after stress (Figure 3—figure supplement 1E), providing a specificity control for any post-ER interaction partners. Conversely, we expected resident ER interactors to be preferentially lost after stress in wild type cells but not in ∆TMED10 cells. Strikingly, the interaction profiles on the stained gels from two independent replicates were essentially unchanged across time points or between wild type versus ∆TMED10 cells (Figure 5D and Figure 5—figure supplement 1C). Only a ~ 24 kD band matching the size of TMED family members was absent from purifications using ∆TMED10 cells.

Of the 2132 proteins represented in the TMT mass spectrometry analysis, very few differed between wild type and ∆TMED10 cells at any of the time points after stress (Figure 5—source data 1), beyond those already discussed above (e.g., compare Figure 5C and E). An important point of comparison is the interactome after 60 min of ER stress, when GFP-PrP* resides entirely in Golgi and post-Golgi compartments in wild type cells (Figure 1E) but entirely in the ER in ∆TMED10 cells (Figure 3—figure supplement 1E). Strikingly, the WT/KO ratios for nearly all GFP-PrP* interactors were very similar before (x-axis) versus after (y-axis) 60 min of ER stress (Figure 5E). Only three proteins showed a high WT/KO ratio preferentially after ER stress: SEP15, PRPF6, and UNC45A. However, two of these (PRPF6 and UNC45A) are nuclear proteins, while the third (SEP15) is a resident ER protein. Thus, none of these are realistic PrP* interactors in the post-ER compartments where PrP* is observed after stress. Furthermore, the differences for PRPF6 and UNC45A are not observed at either 30 or 120 min time points (Figure 5—source data 1), suggesting they are one-off artefacts. The increased WT/KO ratio for SEP15 appears to be due to preferential loss in the KO sample, not an enhanced interaction in WT cells. Thus, no plausible stress-induced post-ER GFP-PrP* interactions could be detected at any time point.

There was an equally striking paucity of interactors that were preferentially reduced or lost upon ER stress in WT cells (Figure 5E). Interacting proteins with reduced WT/KO ratios selectively after stress were either histones, the secreted protein Dermcidin, or the ER-resident protein BiP. Of these, only BiP would seem to be a bona fide interactor and its reduced interaction after acute ER stress preferentially in WT cells is consistent with egress of GFP-PrP* from the ER. However, the reduction was only ~50% despite essentially all GFP-PrP* leaving the ER. Furthermore, other well-validated GFP-PrP* interactors in the ER such as CNX or the TMED family did not show stress-induced reductions in WT/KO ratios that would be expected when GFP-PrP* leaves the ER. These data collectively suggested that the interactome of PrP* is remarkably unchanged during either its constitutive or stress-induced trafficking from the ER to lysosomes via the cell surface.

To investigate this idea more directly, we analyzed both CNX and TMED10 interactions with GFP-PrP* before and after acute ER stress over a 180 min time course. Consistent with the TMT mass spectrometry results, GFP-PrP* associated with both CNX and TMED10 over the entire 180 min time course (Figure 5F). The amount that was associated was completely unchanged for 60 min. Although a reduction in CNX and TMED10 were observed at 180 min, this mirrored the reduced level of GFP-PrP* at this time point due to its lysosomal degradation. By contrast, GFP-PrP only transiently and weakly associated with CNX at the first time point (before ER stress), consistent with its release upon folding and egress to the cell surface. The TMED10 interaction with GFP-PrP declined more slowly as expected for a cargo receptor that accompanies its substrate to at least the Golgi. These results validate the mass spectrometry findings and show that GFP-PrP* retains its associations with factors of the early secretory pathway throughout its trafficking itinerary to the lysosome.

Persistent CNX interaction with PrP* after induction of ER stress was particularly surprising given the earlier finding that this interaction is lost when PrP* exits the ER during stress (Satpute-Krishnan et al., 2014). We believe this discrepancy might be attributed to the different cell types used in the earlier and present studies showing different kinetics of PrP* degradation. It appears that degradation proceeds more rapidly in the cells analyzed previously than the experimental system used here. It may therefore be that at the 30 min time point examined in the earlier study, PrP* was already partially in acidic compartments and being degraded, analogous to the 180 min time point of our experiment. Regardless of the exact reason, our analysis of the PrP* interactome at multiple time points using both quantitative proteomics and direct visualization of interacting partners on stained gels argues strongly that PrP* retains the interactions it makes in the ER at later points during its trafficking.

### Resident ER proteins are associated with cell surface PrP*

**Figure 6.**
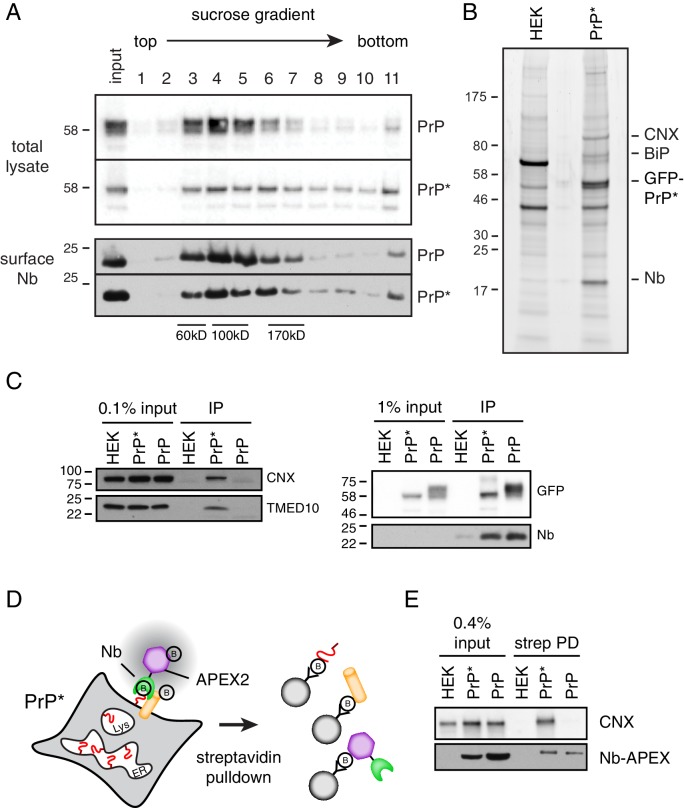
Cell surface PrP* is associated with ER-derived factors. (**A**) Cells expressing GFP-PrP and GFP-PrP* were labeled on ice with saturating levels of extracellular Nb-FLAG, lysed under non-denaturing conditions, and separated by size on a 5–25% sucrose gradient. Fractions were immunoblotted with anti-GFP to detect total cellular GFP-PrP and GFP-PrP*, and anti-FLAG to detect surface-localized Nb complexes. Size standards are estimates based on migration of known proteins on such gradients, which migrate reproducibly from experiment to experiment. (**B**) GFP-PrP*-expressing cells and control HEK293T cells were surface labeled and lysed as in panel A and affinity purified via the FLAG epitope tag on the Nb. Elutions were separated by SDS-PAGE and stained with SYPRO-Ruby, with key proteins indicated on the right. (**C**) Cells expressing GFP-PrP and GFP-PrP* were surface labeled such that equal amounts of anti-GFP Nb-FLAG coated the surface of these two cells. This was accomplished by using saturating (200 nM) Nb for GFP-PrP* cells and 2 nM Nb for GFP-PrP cells. These values were determined in preliminary titration experiments. Surface-localized GFP-PrP and GFP-PrP* were then affinity purified as in panel B, the samples were separated by SDS-PAGE, and subjected to immunoblotting as indicated. The right panel verifies that equal amounts of GFP-PrP and GFP-PrP* were recovered with the Nb in the immunoprecipitates. (**D**) Schematic of the strategy to selectively label proteins near the cell surface population of GFP-PrP* using recombinant anti-GFP Nb-APEX2. Biotinylated proteins are recovered by streptavidin pulldown. (**E**) Cells expressing GFP-PrP and GFP-PrP* were surface labeled such that equal amounts of anti-GFP Nb-APEX2 coated the surface of these two cells, with saturating (200 nM) Nb for GFP-PrP* cells and 0.5 nM Nb for GFP-PrP cells. These values were determined in preliminary titration experiments. After 1 min of biotinylation and quenching, the cells were lysed and the input samples and streptavidin-recovered products were analyzed by immunoblotting.

A major implication of the results in Figure 5 is the entirely unexpected conclusion that GFP-PrP* is associated with normally intracellular resident ER proteins when it arrives at the cell surface. We designed experiments to directly evaluate this idea. Using the ability to selectively tag the minor cell surface population of GFP-PrP* with the tightly binding anti-GFP Nb (Figure 2B), we first evaluated whether surface GFP-PrP* was in larger molecular weight complexes by sucrose gradient sedimentation. As expected from its chaperone interactions in the ER, GFP-PrP* in total lysate migrated far more heterogeneously than GFP-PrP (Figure 6A, top panels): whereas GFP-PrP was primarily in fractions 3–5, substantial amounts of GFP-PrP* were distributed across fractions 6–10 (fraction 11 represents non-solubilized material). Similarly, the distribution of cell surface GFP-PrP* was more heterogeneous than cell surface GFP-PrP (Figure 6A, bottom panels). For example, relatively less GFP-PrP* was seen in fractions 3–5 (where the majority of GFP-PrP was observed), while relatively more GFP-PrP* was seen in fractions 6–10. This difference hinted at the possibility that GFP-PrP* at the cell surface is in protein complex(es) that differ from the mostly uncomplexed GFP-PrP.

**Figure 6—figure supplement 1.**
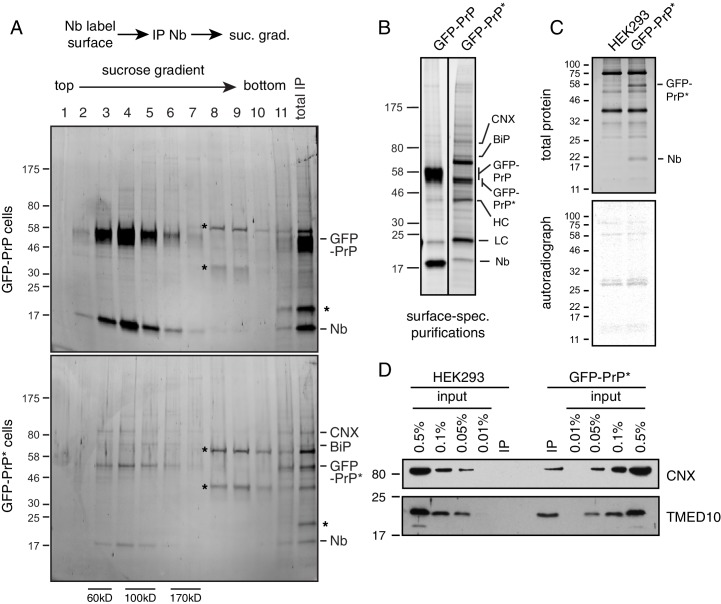
Characterization of cell surface GFP-PrP* complexes. (**A**) Cells expressing GFP-PrP or GFP-PrP* were labeled on ice with saturating levels of Nb-FLAG, washed, and lysed under non-denaturing conditions. The Nb-FLAG complexes were immunopurified via the FLAG epitope tag, eluted under native conditions using FLAG peptide, and separated by size on a 5–25% sucrose gradient. Fractions were analyzed by SDS-PAGE and detected by SYPRO-Ruby stain. Size standards are estimates based on migration of known proteins on such gradients, which migrate reproducibly from experiment to experiment. An aliquot of the total IP is shown in the last lane, with key proteins and the Nb indicated. Asterisks represent the major contaminants found in both samples. As expected, far more GFP-PrP (and associated Nb) is recovered than GFP-PrP*, consistent with the former being present on the surface at ~20 fold higher levels. (**B**) Cells expressing GFP-PrP or GFP-PrP* were surface labeled with Nb-FLAG, washed, lysed under non-denaturing conditions, and affinity purified via the FLAG epitope tag. The elutions were separated by SDS-PAGE and detected with SYPRO-Ruby stain. The two lanes corresponding to GFP-PrP and GFP-PrP* are from the same gel and exposure, with the vertical line indicating where intervening lanes removed. (**C**) GFP-PrP*-expressing cells and control HEK293 cells were labeled on ice with saturating levels of Nb-FLAG and washed. In parallel, GFP-PrP*-expressing cells were radiolabelled with ^35^S-methionine for 1 hr and collected. The Nb-labelled and radiolabelled cells were mixed 1:1, lysed under non-denaturing conditions, and affinity purified via the FLAG epitope tag. Elutions were separated by SDS-PAGE and detected with SYPRO-Ruby stain (top panel) and autoradiography (bottom panel). Note that recovery of radiolabeled proteins was very low and the pattern of radiolabelled proteins was identical in the two samples. This indicates that the unlabeled GFP-PrP* (visible in the stained gel) does not appear to bind radiolabeled proteins after lysis. (**D**) Affinity purified cell surface GFP-PrP* complexes (as in Figure 6B) were compared to serial dilutions of total lysate to estimate the relative amount of CNX and TMED10 recovery. A parallel surface labeling and affinity purification performed on HEK293 cells lacking GFP-PrP* was also analyzed.

**Figure 6—figure supplement 2.**
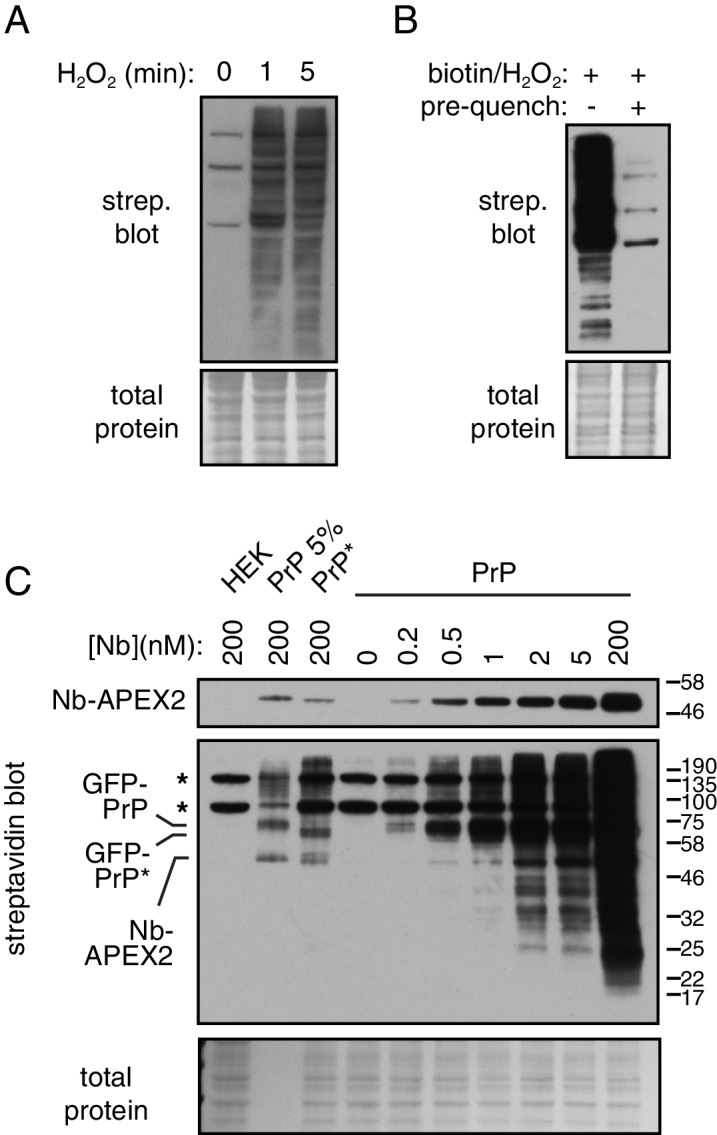
Characterization of APEX2-mediated biotinylation. (**A**) Cells expressing GFP-PrP were surface labeled with 200 nM Nb-APEX2, washed, and incubated with biotin-phenol and H_2_O_2_ for the time indicated prior to quenching. Cell lysates were analyzed for biotinylated proteins by blotting with streptavidin-HRP. The few prominent bands seen at the 0 time point are endogenous biotin-modified proteins. (**B**) Cells expressing GFP-PrP surface labeled with 200 nM Nb-APEX2, washed, incubated with biotin-phenol and H_2_O_2_ for 1 min, then quenched (first lane). Alternatively, the quenching solution was added prior to addition of biotin-phenol and H_2_O_2_ for 1 min (second lane). The cells were then lysed and analyzed for biotinylated proteins by blotting with streptavidin-HRP. Note that if cells are pre-quenched, no biotinylated proteins are observed beyond the few endogenous biotin-modified proteins. (**C**) Cells expressing GFP-PrP and GFP-PrP* were surface labeled with Nb-APEX2 added at the indicated concentrations to the medium, washed, then incubated with biotin-phenol and H_2_O_2_ for 1 min. For comparison, HEK293 cells are shown as a negative control. The positions of the most prominently labeled proteins (Nb-APEX2 and its target antigen) are indicated.

Using an affinity tag on the Nb, we selectively purified the surface population of GFP-PrP* after first washing away all excess extracellular Nb. For comparison, we performed the same experiment with GFP-PrP. Lysis conditions were chosen such that GFP-PrP* is efficiently solubilized (Figure 2C) while still maintaining both Nb-GFP interactions (Figure 2D) and putative PrP* interactions (Figure 6A). Sucrose gradient analysis of the eluates from these surface-specific purifications showed that GFP-PrP migrates as an uncomplexed protein without any appreciable co-purifying proteins (Figure 6—figure supplement 1A). By contrast, GFP-PrP* co-purified with proteins of ~75 and 90 kD that were identified by mass spectrometry and immunoblotting as BiP and CNX, respectively (Figure 6—figure supplement 1A). These proteins seemed to migrate in somewhat different fractions suggesting that they were either in separate complexes with GFP-PrP* or might have dissociated during elution or gradient fractionation.

Multiple independent large-scale surface purifications of cell surface GFP-PrP* were analyzed by mass spectrometry and verified to contain BiP, CNX, TMED family members, and lesser amounts of other ER-resident chaperones like GRP94 (Figure 6B and Figure 6—figure supplement 1B). These surface interactions were validated by immunoblotting to be specific for GFP-PrP* when compared with equal amounts of GFP-PrP purified selectively from the surface (Figure 6C). Separate experiments ensured that the Nb does not dissociate from GFP-PrP* and rebind intracellular GFP-PrP* molecules after lysis (Figure 2—figure supplement 1B), consistent with the Nb’s extremely slow off rate from GFP. Furthermore, post-lysis mixing with a radiolabeled cell lysate did not recover any radiolabeled proteins selectively in the samples containing Nb-GFP-PrP* complexes (Figure 6—figure supplement 1C). This argues against chaperone interactions with surface GFP-PrP* occurring after lysis.

Quantification relative to serial dilutions of total lysate indicates that ~ 0.1% of total CNX was recovered with surface GFP-PrP* (Figure 6—figure supplement 1D). Given a purification efficiency of ~20% and the fact that ~ 4.4% of all GFP-PrP* is on the surface, we can estimate that ~ 0.9% of total cellular GFP-PrP* is recovered in the experiment. Because CNX is a very abundant protein, recovery of ~0.1% of total cellular CNX with ~0.9% of total GFP-PrP* would make them of comparable abundance in the purified complex, consistent with what is observed on stained gels. GFP-PrP* is also associated with other abundant chaperones and cargo receptors at similar or somewhat lower levels; hence, the sum of these interactors would be comparable to the abundance of surface GFP-PrP*. This would be consistent with the conclusion from time-resolved mass spectrometry results (Figure 5E) that GFP-PrP*’s interactome in post-ER compartments is essentially unchanged from its ER interactome.

As a final orthogonal test of the idea that surface GFP-PrP* retains its intracellular interaction partners, we used a selective enzymatic labeling strategy to biotinylate GFP-PrP* neighbors on intact live cells (Lam et al., 2015; Rhee et al., 2013). We prepared a recombinant Nb fusion with the peroxidase APEX2 and used purified Nb-APEX2 to selectively decorate the surface population of GFP-PrP* or GFP-PrP. When the APEX substrate biotin-phenol is added to the extracellular medium, it is converted by APEX2 to a highly reactive membrane-impermeable intermediate that biotinylates nearby proteins (Figure 6D). Preliminary experiments using GFP-PrP cells identified the minimum saturating concentrations of Nb-APEX and showed that proximity labeling could be achieved within 1 min (Figure 6—figure supplement 2A). Importantly, quenching conditions were identified after which no detectable labeling could be observed (Figure 6—figure supplement 2B).

After decorating GFP-PrP* cells with Nb-APEX2, removing the excess, labeling with biotin-phenol for 1 min, and quenching, the biotinylated proteins were recovered via immobilized streptavidin. CNX was recovered as detected by immunoblotting (Figure 6E). When GFP-PrP cells were treated in an identical manner under conditions of equal Nb-APEX2 surface density (Figure 6—figure supplement 2C), CNX was not effectively recovered in the streptavidin pulldowns (Figure 6E). Thus, in live and intact cells, surface GFP-PrP* is selectively adjacent to CNX, which has apparently accompanied it from the ER on the way to lysosomes.

### Acute misfolding at the cell surface does not trigger degradation

The sustained interaction with intracellular factors during its trafficking itinerary, together with the apparent lack of any new interactions at post-Golgi compartments, suggested the hypothesis that the intracellular factors are a critical cue for PrP*’s rapid removal from the cell surface. In this model, both PrP and PrP* arrive at the cell surface, but one or more of the factors PrP* has retained from inside the cell triggers its endocytosis and lysosomal degradation. The large number of factors, their critical roles in intracellular homeostasis and trafficking, and the likelihood of pleiotropic consequences upon their disruption posed major challenges to testing this idea. For example, deleting TMED10 precludes ER exit of PrP*, preventing a straightforward test of whether it has a crucial role in PrP* endocytosis.

To circumvent these obstacles, we designed a generic strategy to test the functional importance of retained intracellular interaction partners for rapid removal of misfolded GPI-anchored proteins from the cell surface. We reasoned that if these factors are important at the surface, then acute misfolding of a GPI-anchored protein only after it had reached the surface should preclude its recognition and endocytosis. By contrast, if a separate surface surveillance system (which has eluded our proteomic searches) is instead critical, then acute misfolding would be recognized regardless of the absence of retained intracellular factors. Our approach to test this idea relied on the observation that the folded state of a mutant FKBP12 (hereafter simply FKBP*) is stabilized by a bound ligand called Shield1 (Banaszynski et al., 2006; Egeler et al., 2011). We could then allow the GPI-anchored FKBP* reporter to reach the surface of cells grown in the presence of Shield1, mark only the surface population via extracelluar Nb, then withdraw Shield1 and follow the fate of misfolded surface FKBP*.

**Figure 7.**
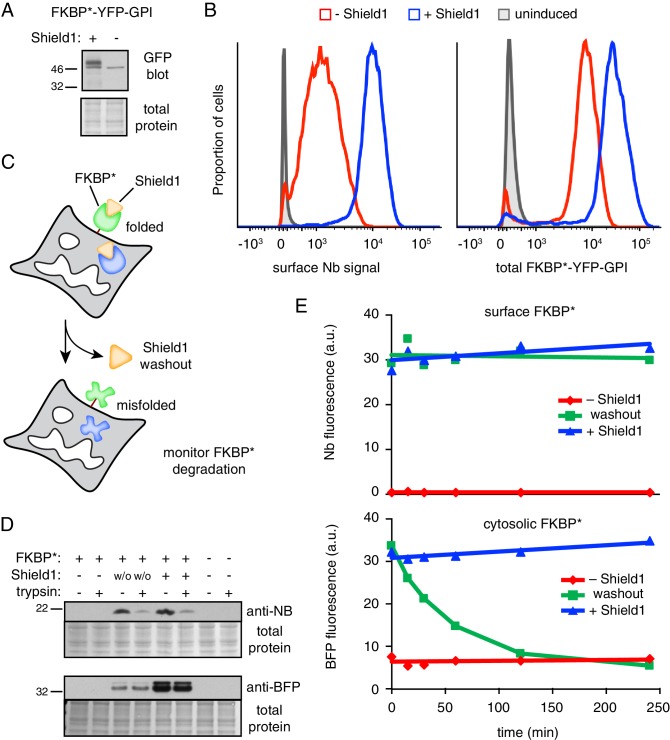
GPI-anchored protein acutely misfolded at the cell surface is not degraded. (**A**) Cells transiently transfected with FKBP*-YFP-GPI for 48 hr were grown in the presence or absence of 2 µM Shield1, lysed, and immunoblotted using anti-GFP (which also detects YFP). (**B**) Cells stably expressing inducible FKBP*-YFP-GPI grown with or without Shield1 were incubated on ice with 200 nM Alexa647-conjugated anti-GFP Nb and analyzed by flow cytometry for Nb fluorescence (left) or total YFP fluorescence (right). (**C**) Schematic of experimental strategy for simultaneousy monitoring the fate of inducibly misfolded FKBP* in the cytosol and on the cell surface. The surface FKBP* is tagged with YFP and the cytosolic FKBP* is tagged with tagBFP. (**D**) Cells transiently transfected with FKBP*-YFP-GPI and cytosolic FKBP*-BFP for 48 hr were grown in the presence or absence of 2 µM Shield1. Following labeling on ice with Nb-FLAG, cells were washed and incubated for a further two hours at 37°C. During this incubation period, half of the cells previously grown in Shield1 were subjected to washout (w/o) by omitting Shield1 and further supplementing the medium with excess recombinant FKBP* to act as a molecular ‘sink’ to capture any residual Shield1. After the incubation period, half of the cells were treated with extracellular trypsin to digest surface-exposed proteins before inactivating the trypsin. Following lysis, Nb-FLAG and cytosolic FKBP*-BFP were detected by immunoblotting. (**E**) Cells transiently transfected with FKBP*-YFP-GPI and cytosolic FKBP*-BFP for 48 hr were grown in the presence or absence of 2 µM Shield1. In a subset of cells, Shield1 was withdrawn as in panel D for varying periods of time, after which surface-localized FKBP*-YFP-GPI was stained with Alexa647-Nb. Levels of cytosolic FKBP*-BFP and surface-localized FKBP*-YFP-GPI were monitored via flow cytometry. 2.5 µg/ml Brefeldin A was included in the medium during the time course to prevent export of newly-synthesized protein.

We first analyzed the fate of a YFP-tagged GPI-anchored version of FKBP* (FKBP*-YFP-GPI) in the absence and presence of Shield1. As expected from the study of GFP-PrP* and other GPI-anchored proteins, FKBP*-YFP-GPI stabilized by Shield1 was primarily trafficked to the surface, while FKBP*-YFP-GPI was primarily intracellular in the absence of Shield1. This was evident from Golgi-dependent glycan maturation only in the presence of Shield1 (Figure 7A), markedly reduced surface staining by extracellular Nb selectively in the absence of Shield1 (Figure 7B, left), and direct visualization by fluorescence microscopy (data not shown). The overall level of FKBP*-YFP-GPI was lower at steady state in the absence of Shield1, consistent with its constitutive degradation (Figure 7B, right). Importantly, acute ER stress of FKBP*-YFP-GPI expressing cells grown in the absence of Shield1 led to a stimulation of extracellular Nb uptake (Figure 7—figure supplement 1A) and degradation (Figure 7—figure supplement 1B). Thus, as with GFP-PrP*, Shield1-lacking FKBP*-YFP-GPI is primarily retained intracellularly and degraded via a cell surface trafficking itinerary that is stimulated by acute ER stress. By contrast, Shield1-bound FKBP*-YFP-GPI behaves like GFP-PrP and is trafficked appropriately to the cell surface where it resides at steady state.

**Figure 7—figure supplement 1.**
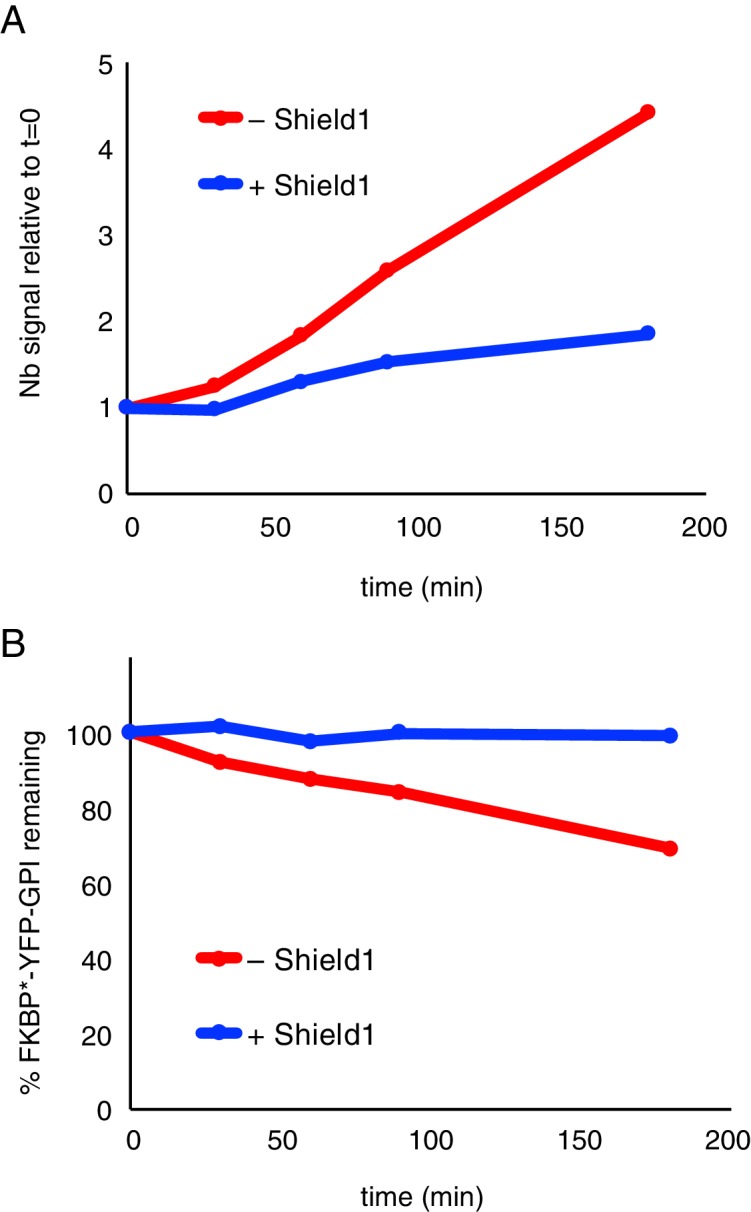
Characterization of FKBP*-YFP-GFP cells. (**A**) Cells stably expressing inducible FKBP*-YFP-GPI grown with or without Shield1 were allowed to internalize Alexa647-Nb from the culture medium in the presence of thapsigargin-induced ER stress and 100 µg/ml cycloheximide. The graph depicts the median fluorescence intensity of internalized nanobody at each time point relative to time 0, as measured by flow cytometry. (**B**) Total YFP fluorescence was measured in cells over a time course experiment as in panel A. The graph depicts median fluorescence intensity as a percentage of time 0.

To test the effect of acute misfolding at the cell surface (Figure 7C), cells expressing FKBP*-YFP-GPI were cultured in the presence of Shield1, the surface population was marked with extracellular Nb, and the Shield1 was withdrawn. As expected, little or no Nb was recovered from FKBP*-YFP-GPI cells grown without Shield1 (Figure 7D, lane 1) or from cells lacking FKBP*-YFP-GPI (Figure 7D, lane 7). By contrast, clear Nb binding was evident in Shield1-grown FKBP*-YFP-GPI cells (Figure 7D, lane 5). This Nb was localized at the surface as evidenced by its digestion by extracellular trypsin. Strikingly, essentially all of the initially bound Nb was still present 2 hr after Shield1 withdrawal, and these Nb molecules were still at the surface (and hence, trypsin accessible) to the same level as that observed before withdrawal (Figure 7D, lanes 3 and 4). Importantly, a cytosolic FKBP* tagged with blue fluorescent protein (FKBP*-BFP) expressed in the same cells was fully protected from extracellular trypsin, and mostly degraded 2 hr after Shield1 withdrawal. This cytosolic reporter provides an internal control verifying the efficacy of Shield1 withdrawal and the selectivity of trypsin digestion for only surface exposed proteins. Thus, acute destabilization of FKBP*-GFP-GPI at the cell surface by Shield1 withdrawal does not cause its internalization from the cell surface or degradation in lysosomes.

To verify this conclusion and to exclude any possibility that pre-binding the Nb somehow prevented FKBP*-YFP-GPI internalization, we performed the experiment in a different way. Using cells grown in Shield1 that co-express FKBP*-BFP in the cytosol and FKBP*-YFP-GPI at the cell surface, we withdrew Shield1 for different periods of time, stained the surface population of FKBP*-YFP-GPI using fluorescent Nb, and analyzed the cells by flow cytometry. As expected, the FKBP*-BFP in the cytosol was rapidly degraded after Shield1 withdrawal to the same low level observed when cells are grown in the absence of Shield1 (Figure 7E). Additionally, retaining Shield1 during the monitoring period resulted in no degradation. In the case of FKBP*-YFP-GPI, the amount on the surface remained unchanged during the time course regardless of whether Shield1 was maintained or withdrawn. This level on the surface was markedly higher than the steady-state surface level seen when cells are cultured in the absence of Shield1.

Taken together, these results show that when FKBP*-YFP-GPI misfolds at the time of its biogenesis at the ER, it is degraded. By contrast, when it is allowed to go to the surface as a folded protein, then misfolds, the surface population fails to be recognized as aberrant and remains there as if it were folded. This strongly indicates that not only is misfolding initially recognized in the ER, but the misfolding-dependent interactions made in the early secretory pathway need to be retained for cells to recognize protein misfolding at the cell surface. Thus, FKBP*-YFP-GPI that misfolds in the ER and arrives at the surface is distinct from FKBP*-YFP-GPI that misfolds at the plasma membrane: the former is associated with intracellular factors, while the latter cannot do so. This biochemical difference between these two situations appears to be a critical determinant of the misfolded protein’s ultimate fate.

## Discussion

We have systematically analyzed the trafficking itinerary and physical interactions made by PrP*, a model misfolded GPI-anchored protein, during its degradation in cultured mammalian cells. Our analysis leads to a number of new insights. First, essentially all PrP* transits the cell surface on its way to lysosomes for degradation. Second, PrP* has a short residence time on the cell surface unlike properly folded PrP, explaining why only a tiny proportion of PrP* is detected on the surface at steady-state. Third and most unexpectedly, PrP* arrives at the cell surface in complex with the chaperones and cargo receptors that it engaged during its initial misfolding in the ER lumen. Finally, using a GPI-anchored protein whose misfolding could be temporally controlled in trans, we find that persistent interaction with intracellular factors is a key requirement for cellular recognition of misfolding at the cell surface. Each of these new insights has a number of important implications for our understanding of quality control in the secretory pathway and the post-ER roles of factors traditionally restricted to the early secretory pathway.

Earlier studies of both natural (Ashok and Hegde, 2009) and artificial (Satpute-Krishnan et al., 2014) misfolding mutants of PrP had established that lysosomal degradation is a major pathway for their turnover. This conclusion was generalized for other GPI-anchored proteins in mammalian cells (Satpute-Krishnan et al., 2014) and yeast (Sikorska et al., 2016). However, the route to lysosomes, while known to involve transit through the Golgi, was not clear. A major conclusion from our quantitative analysis is that the majority of PrP* samples the cell surface prior to lysosomal degradation. Earlier qualitative antibody uptake experiments had hinted at this possibility (Satpute-Krishnan et al., 2014), but the extent of flux through this route was not determined. It is not known whether this conclusion applies uniformly to all GPI-anchored proteins, all types of mutants, and other organisms (e.g., yeast). However, our findings that FKBP*, an unrelated misfolded protein, also accesses the cell surface argues for the generality of our findings.

The remarkable implication of this conclusion is that cells transiently expose misfolded proteins to the extracellular environment. This is surprising given the extensive quality control and retention pathways that eukaryotes have evolved to explicitly avoid this fate. It seems unlikely that other routes to the lysosome are not available to the cell. Indeed, inhibition of endocytosis via AP2 or dynamin perturbation, while detectably increasing the amount of PrP* at the cell surface, does not fully trap all PrP* at the cell surface and does not strongly impair degradation. While this finding is difficult to interpret cleanly given the pleiotropic consequences of these manipulations for other trafficking pathways and perhaps lysosome function, it does illustrate that cells have alternative degradative options for GPI-anchored proteins including ERAD (Sikorska et al., 2016), and possibly direct Golgi-to-lysosome traffic (Coutinho et al., 2012) or autophagy of the ER (Forrester et al., 2019). It is therefore worth considering whether the favored surface route provides an unappreciated benefit. One attractive idea is that perhaps surface exposure, either of misfolded proteins directly or of their associated chaperones (see below), is used to judge a cell’s health or stress status by other cells in a metazoan organism (Yu et al., 2015).

The misfolded GPI-anchored protein that arrives at the cell surface is complexed with chaperones and cargo receptors. Although we identified several proteins in cell surface PrP* complexes, it is unclear whether they are all part of a single complex or instead represent heterogeneity of complexes. Regardless, their association provides a satisfying explanation for how a grossly misfolded protein can navigate the secretory pathway without risk of aggregation or inappropriate interactions. The chaperones may remain tightly bound after PrP* leaves the ER due to the absence of co-factors that drive their recycling, thereby preventing further attempts at folding. The tight binding of chaperones, particularly the lectin CNX, nicely explains the otherwise puzzling observation that PrP*’s glycans do not get trimmed or modified during its transit through the Golgi. This is presumably why the vast majority of PrP* isolated from the cell surface still has immature glycans while PrP’s glycans are mature.

Our attempts to directly detect endogenous chaperones and cargo receptors on the cell surface have faced substantial challenges due to the extremely low proportion of these normally ER-resident proteins on the surface combined with the limitations of available antibodies. Only ~0.5% of cellular CNX and TMED10 molecules are engaged with PrP* on the surface (Figure 6C; Figure 6—figure supplement 1D), and possibly even less when PrP* is not being expressed. Monitoring this tiny population requires reagents that bind with exquisitely high affinity and specificity, such as the anti-GFP nanobody that enabled us to detect surface-localised GFP-PrP*. Unfortunately, the available antibodies against the non-cytosolic domains of endogenous ER-resident proteins do not provide sufficient signal-to-noise to reliably detect them at the cell surface or follow the fate of these minor populations. Achieving this goal will probably require development of new reagents (e.g., high affinity nanobodies) or tagging the factors in the genome in a way that does not disrupt function or trafficking.

It is unknown what ultimately happens to chaperones and cargo receptors that accompany PrP* on its degradative route. The simplest model is that they are degraded in the lysosome and are essentially serving a terminal function. This has not been possible to test with lysosomal inhibitors because the proportion that would be stabilized is very small. Regardless, our data tracking PrP* interactions over a 180 min time course suggests that no appreciable dissociation occurs before delivery to the late endocytic pathway. An extensive but heterogeneous literature has reported chaperones (Altmeyer et al., 1996; Calderwood et al., 2007a; Okazaki et al., 2000; Wiest et al., 1997; Zhang et al., 2010) or cargo receptors (Blum and Lepier, 2008) in post-ER compartments including the cell surface and extracellular space. While the functional relevance of these observations has largely remained unclear, their elevated exposure has sometimes been associated with cancer cells (Arap et al., 2004; Shin et al., 2003; Tsai et al., 2015). Because cancer cells have a high mutation burden and are thought to express numerous misfolded proteins, elevated surface chaperones might reflect their involvement in degradation pathway(s) like the one described in this study.

As noted above, one role of retaining chaperone and cargo receptor interactions may be to shield the misfolded proteins from inappropriate interactions. Our acute surface misfolding experiments argues that a second critical function is to cue endocytosis and degradation. At present, we cannot determine which of the PrP* surface interaction partners is critical for this endocytosis function. One model is that cells express a transmembrane ‘receptor’ for certain chaperones, and engagement of this receptor triggers endocytosis. Indeed, putative receptors that recognize chaperones have been described (Calderwood et al., 2007b), although evidence for such an interaction did not emerge from our systematic analysis of PrP* interactions. A related model involves direct recognition of transmembrane chaperones or cargo receptors associated with PrP*. For example, TMED family members have cytosolic tails that could be recognized for endocytosis at the plasma membrane. The currently ill-defined complex of TMEDs that associate with PrP* and the presence of multiple family members makes testing this idea challenging, but it remains an important future goal.

It is striking that a GPI-anchored protein that misfolds at the cell surface goes unrecognized. In recent years, a growing number of studies have provided evidence for quality control at the plasma membrane. In these cases, the recognition factors are all in the cytosol and the clients are all integral membrane proteins with cytosolically exposed regions (Apaja et al., 2013; Apaja et al., 2010; Okiyoneda et al., 2018; Okiyoneda et al., 2010; Zhao et al., 2013). Few studies have rigorously limited the misfolding to an extracellular domain and investigated whether cells can recognize the problem. Given the constant exposure of cell surface proteins to the changing and often harsh extracellular environment, one might have predicted the existence of a mechanism for detection of misfolded extracellular domains. While our data do not exclude such a system, its mode of recognition must differ from that used in the cytosol given the different behavior of Shield1-lacking FKBP* in the two compartments. Given that PrP* is associated with intracellular factors at the surface, we posit that GPI-anchored proteins as a class rely on this mechanism for their recognition at the plasma membrane.

## Materials and methods

**Key resources table:** 

Reagent type (species) or resource	Designation	Source or reference	Identifiers	Additional information
Cell line (*Homo sapiens*)	HEK293T	ATCC CRL-3216	RRID:CVCL_0063	
Cell line (*H. sapiens*)	HEK293 TRex Flp-in	Invitrogen	RRID:CVCL_U427	
Cell line (*H. sapiens*)	HEK293 TRex GFP-PrP*	This paper		GFP-PrP* (see below) integrated into FRT site of HEK293 Trex Flp-in cell line
Cell line (*H. sapiens*)	HEK293 TRex GFP-PrP	This paper		GFP-PrP (see below) integrated into FRT site of HEK293 Trex Flp-in cell line
Cell line (*H. sapiens*)	HEK293 TRex GFP-PrP* TMED10 KO	This paper		Disruption of TMED10 by CRISPR/Cas9 in GFP-PrP* cell line
Cell line (*H. sapiens*)	HEK293 TRex GFP-PrP TMED10 KO	This paper		Disruption of TMED10 by CRISPR/Cas9 in GFP-PrP cell line
Cell line (*H. sapiens*)	HEK293 TRex FKBP*-YFP-GPI	This paper		FKBP*-YFP-GPI (see below) integrated into FRT site of HEK293 Trex Flp-in cell line
Antibody	rabbit polyclonal anti-GFP	Stefanovic and Hegde, 2007		(1:5000)
Antibody	rabbit polyclonal anti-RFP	Stefanovic and Hegde, 2007		(1:3000)
Antibody	mouse monoclonal anti-FLAG (M2)-HRP conjugated	Sigma	Cat #A8592, RRID:AB_439702	(1:10000)
Antibody	rabbit polyclonal anti-TMED10 C-terminus	Satpute-Krishnan et al., 2014		(1:1000)
Antibody	rabbit polyclonal anti-TMED2	Jenne et al., 2002		(1:6000)
Antibody	rabbit polyclonal anti-TMED10 lumenal domain	Jenne et al., 2002		(1:5000)
Antibody	rabbit polyclonal anti-TMED7	Jenne et al., 2002		(1:5000)
Antibody	rabbit polyclonal anti-TMED9	Jenne et al., 2002		(1:3000)
Antibody	rabbit polyclonal anti-CNX N-terminus	Enzo	Cat #ADI-SPA-865, RRID:AB_10618434	(1:2000 – 1:5000)
Antibody	rabbit polyclonal anti-CNX C-terminus	Enzo	Cat #ADI-SPA-860, RRID:AB_10616095	(1:2000)
Antibody	rabbit polyclonal anti tagRFP (also recognizes tagBFP)	evrogen	Cat #AB233, RRID:AB_2571743	(1:2000)
Antibody	mouse monoclonal anti-alpha adaptin (AP2)	BD Biosciences	Cat #610502, RRID:AB_397868	(1:3000)
Antibody	mouse monoclonal anti-Transferrin receptor	Invitrogen	Cat #136800, RRID:AB_2533029	(1:500)
Recombinant DNA reagent	pRSET-Nanobody-3x FLAG-His	This paper		Nanobody sequence from Addgene plasmid #49172
Recombinant DNA reagent	pX330-TMED10 sgRNA2	This paper		sgRNA targeting TMED10
Recombinant DNA reagent	pX330-TMED10 sgRNA3	This paper		sgRNA targeting TMED10
Recombinant DNA reagent	pcDNA3-HA-TMED10	This paper		Human TMED10 with HA tag following signal sequence
Recombinant DNA reagent	pcDNA3-HA-TMED10 si2R	This paper		HA-tagged human TMED10 as above with silent mutations to confer resistance to Stealth RNAi TMED10HSS 145904
Recombinant DNA reagent	pcDNA3-HA-RFP-TMED10	This paper		Human TMED10 with HA tag and RFP following signal sequence
Recombinant DNA reagent	pcDNA3-HA-TMED10 ΔCC	This paper		Human HA-TMED10 lacking coiled-coil domain (amino acids 130–183)
Recombinant DNA reagent	pcDNA3-HA-RFP-TMED10 ΔGOLD	This paper		Human HA-RFP-TMED10 with GOLD domain deleted (residues 41–129 of original protein)
Recombinant DNA reagent	pcDNA3-HA-RFP-TMED10 ΔCC	This paper		Human HA-RFP-TMED10 lacking coiled-coil domain (amino acids 130–183)
Recombinant DNA reagent	FKBP12-YFP-GPI	Sengupta et al., 2015		
Recombinant DNA reagent	FKBP*-YFP-GPI	This paper		F36V and L106P mutations introduced into FKBP12-YFP-GPI
Recombinant DNA reagent	pcDNA5-FRT/TO-FKBP *-YFP-GPI	This paper		FKBP*-YFP-GPI subcloned into pcDNA5-FRT/TO for stable inducible expression
Recombinant DNA reagent	FKBP*-BFP	This paper		FKBP* followed by BFP for cytosolic expression
Recombinant DNA reagent	pET15b-HisFKBP*12 F36V	Addgene	Cat #73180, RRID:Addgene_73180	
Recombinant DNA reagent	FusionRed-Dynamin S45N	Almeida-Souza et al., 2018		Dominant-negative dynamin mutant
Recombinant DNA reagent	pcDNA3-3xmyc-TMED2	This paper		Human TMED2 with 3xmyc tag following signal sequence.
Recombinant DNA reagent	pRSET-Nanobody- APEX2-3xFLAG-His	This paper		Nanobody-APEX2-FLAG for bacterial expression. APEX2 sequence from Addgene plasmid #49386
Recombinant DNA reagent	mGFP1-N1	Clontech		
Recombinant DNA reagent	pcDNA5-FRT/TO- EGFP-PrP*	This paper		C179A mutant of hamster PrP with bovine prolactin signal sequence; EGFP @ unique Bsu36I site downstream of signal sequence.
Recombinant DNA reagent	pcDNA5-FRT/TO- EGFP-PrP	This paper		Wild-type human PrP, with EGFP @ unique Bsu36I site downstream of signal sequence.
Sequence-based reagent	CRISPR: TMED10 sgRNA2	IDT		TAACGGAAAAGGGCCGCGCC
Sequence-based reagent	CRISPR: TMED10 sgRNA3	IDT		GCAGCAACGCTAACGGAAAA
Sequence-based reagent	siRNA: nontargeting control	Dharmacon	D-001810–10	
Sequence-based reagent	siRNA: AP2 (alpha-adaptin)	Dharmacon		gift of B. Nichols lab (MRC-LMB)
Sequence-based reagent	siRNA: nontargeting control	Thermo Fisher	4390843	Silencer Select
Sequence-based reagent	siRNA: TMED1 (a)	Thermo Fisher	s21699	Silencer Select
Sequence-based reagent	siRNA: TMED1 (b)	Thermo Fisher	s21700	Silencer Select
Sequence-based reagent	siRNA: TMED2 (a)	Thermo Fisher	s21570	Silencer Select
Sequence-based reagent	siRNA: TMED2 (b)	Thermo Fisher	s21571	Silencer Select
Sequence-based reagent	siRNA: TMED3 (a)	Thermo Fisher	s23799	Silencer Select
Sequence-based reagent	siRNA: TMED3 (b)	Thermo Fisher	s23800	Silencer Select
Sequence-based reagent	siRNA: TMED4 (a)	Thermo Fisher	s48156	Silencer Select
Sequence-based reagent	siRNA: TMED4 (b)	Thermo Fisher	s48157	Silencer Select
Sequence-based reagent	siRNA: TMED5 (a)	Thermo Fisher	s27202	Silencer Select
Sequence-based reagent	siRNA: TMED5 (b)	Thermo Fisher	s27203	Silencer Select
Sequence-based reagent	siRNA: TMED6 (a)	Thermo Fisher	s44861	Silencer Select
Sequence-based reagent	siRNA: TMED6 (b)	Thermo Fisher	s44862	Silencer Select
Sequence-based reagent	siRNA: TMED7 (a)	Thermo Fisher	s27238	Silencer Select
Sequence-based reagent	siRNA: TMED7 (b)	Thermo Fisher	s27239	Silencer Select
Sequence-based reagent	siRNA: TMED9 (a)	Thermo Fisher	s29353	Silencer Select
Sequence-based reagent	siRNA: TMED9 (b)	Thermo Fisher	s29354	Silencer Select
Sequence-based reagent	siRNA: TMED10	Thermo Fisher	HSS145904	Stealth siRNA
Sequence-based reagent	siRNA: negative control	Thermo Fisher	46–2001	Stealth siRNA
Peptide, recombinant protein	Nanobody-FLAG-His	This paper		purified from *E. coli* (BL21) pLysS cells using immobilized metal affinity chromatography
Peptide, recombinant protein	Nanobody-FLAG-APEX2	This paper		purified from *E. coli* (BL21) pLysS cells using immobilized metal affinity chromatography
Peptide, recombinant protein	FKBP12 (F36V)	Egeler et al., 2011		purified from *E. coli* (BL21) pLysS cells using immobilized metal affinity chromatography
Chemical compound, drug	Thapsigargin	Sigma	Cat #T9033	
Chemical compound, drug	Bafilomycin A1	Sigma	Cat #B1793	
Chemical compound, drug	Brefeldin A	Invitrogen	Cat #B7450	
Chemical compound, drug	Shield1	Clontech/Takara	Cat #632189	
Chemical compound, drug	Cycloheximide	Sigma	Cat #C4859	
Chemical compound, drug	Alexa Fluor546 NHS Ester	Invitrogen	Cat #A20002	
Chemical compound, drug	Alexa Fluor647 NHS Ester	Invitrogen	Cat #A37566	
Chemical compound, drug	TMT labeling reagents	Thermo Fisher	Cat #90110	
Chemical compound, drug	biotin-phenol	Iris Biotech	Cat #LS-3500	
Other	GFP-trap	Chromotek	Cat #gta-20	
Other	anti-FLAG M2 affinity resin	Sigma	Cat #A2220	
Other	Streptavidin-HRP	Thermo Fisher	Cat #43–4323	
Other	Streptavidin T1 Dynabeads	Invitrogen	Cat #65601	

### Cell culture

Flp-in T-Rex HEK293 cells (Invitrogen) and HEK293T cells were cultured in DMEM supplemented with 10% fetal bovine serum (FBS). In cases where the cells contained a stably expressed doxycycline-inducible protein, tetracycline-free FBS was used as well as 10 μg/ml blasticidin and 100 μg/ml hygromycin. Cells inducibly expressing GFP-PrP, GFP-PrP*, and FKBP*-YFP-GPI were made by integrating the respective expression cassettes into the FRT site of Flp-in T-Rex HEK293 cells by Flp recombination (Invitrogen). 10 ng/ml doxycycline was used for induction of the integrated gene at the FRT site. All cell lines were checked for mycoplasma contamination using the MycoAlert Mycoplasma Detection Kit (Lonza) and found to be negative. Cell line identities were verified by a combination of an integrated FRT site and doxycycline inducibility (distinctive to Flp-in T-Rex cells), by antibiotic resistance markers, by homogeneous fluorescent reporter expression, and by immunoblotting for the product of knockout alleles. Further genetic analysis was not performed. Transient transfections were performed using TransIT-293 (Mirus Bio), according to manufacturer’s instructions. For flow cytometry experiments in which expression constructs did not contain a fluorescent protein, RFP was co-transfected as a transfection marker so that only transfected cells could be analyzed. siRNA silencing was performed with Lipofectamine RNAiMAX (Life Technologies) as directed by the manufacturer. AP2 siRNA was obtained from Dharmacon and used at 20 nM. Silencing was allowed to proceed for 96 hr after transfection prior to analysis. Silencer Select siRNAs against TMED family proteins were obtained from ThermoFisher, and used at 10 nM. Silencing was allowed to proceed for 72 hr after transfection prior to analysis. Gene disruption of TMED10 by CRISPR was performed as described (Ran et al., 2013) with the following guide RNA sequences: TAACGGAAAAGGGCCGCGCC and GCAGCAACGCTAACGGAAAA. Clones were verified by immunoblotting to be disrupted for TMED10, and knockout cell lines with either guide behaved the same way.

### Constructs

**Table:** 

Plasmid name	Purpose	Brief description	Backbone vector	Promoter
pRSET-Nanobody-3xFLAG-His	bacterial expression	3xFLAG-tagged anti-GFP nanobody	pRSET	T7
pX330-TMED10 sgRNA2	mammalian cell expression	sgRNA targeting TMED10 (TAACGGAAAAGGGCCGCGCC)	pX330 (ΔCas9)	U6
pX330-TMED10 sgRNA3	mammalian cell expression	sgRNA targeting TMED10 (GCAGCAACGCTAACGGAAAA)	pX330 (ΔCas9)	U6
pcDNA3-HA-TMED10	mammalian cell expression	Human TMED10 with HA tag following signal sequence	pcDNA3.1 (+) zeo	CMV
pcDNA3-HA-TMED10 si2R	mammalian cell expression	HA-tagged human TMED10 as above with silent mutations to confer resistance to Stealth RNAi TMED10HSS145904	pcDNA3.1 (+) zeo	CMV
pcDNA3-HA-RFP-TMED10	mammalian cell expression	Human TMED10 with HA tag and RFP following signal sequence	pcDNA3.1 (+) zeo	CMV
pcDNA3-HA-TMED10 ΔCC	mammalian cell expression	Human TMED10 lacking coiled-coil domain (amino acids 130–183); HA-tagged	pcDNA3.1 (+) zeo	CMV
pcDNA3-HA-RFP-TMED10 ΔGOLD	mammalian cell expression	GOLD domain deleted (residues 41–129 of original protein); HA- and RFP-tagged	pcDNA3.1 (+) zeo	CMV
pcDNA3-HA-RFP-TMED10 ΔCC	mammalian cell expression	Human TMED10 lacking coiled-coil domain (amino acids 130–183); HA- and RFP-tagged	pcDNA3.1 (+) zeo	CMV
FKBP*12(F36V/L106P)-YFP-GPI	mammalian cell expression	FKBP*12 containing destabilizing mutations, followed by YFP and GPI- anchor signal. For transient expression.	Derived from FKBP12-YFP-GPI (Sengupta et al., 2015) provided by J. Lippincott-Schwartz	
FKBP*12(F36V/L106P)-BFP	mammalian cell expression	FKBP*12 containing destabilizing mutations, followed by BFP	pcDNA3.1 (+) zeo	CMV
pcDNA5-FRT/TO-YFP- FKBP*12(F36V/L106P)-GPI	mammalian cell expression	FKBP*12 containing destabilizing mutations, followed by YFP and GPI- anchor signal. For stable- inducible expression.	pcDNA5/FRT/TO	CMV Tet-on
pET15b-HisFKBP*12 F36V	bacterial expression	Recombinant FKBP*12 to bind excess Shield1 during washout.	Created by T. Wandless (Addgene Plasmid #73180)	
FusionRed-Dynamin S45N	mammalian cell expression	Dominant-negative dynamin mutant	Gift from H. McMahon's lab (MRC-LMB, Cambridge)	
pcDNA3-3xmyc-TMED2	mammalian cell expression	Human TMED2 with 3xmyc tag following signal sequence.	pcDNA3.1 (+) zeo	CMV
nanobody APEX2	bacterial expression	nanobody-APEX2-FLAG for bacterial expression	pRSET	T7
mGFP1-N1	mammalian cell expression	cytosolic GFP	pmGFP	
pcDNA5-FRT/TO-EGFP-PrP*	mammalian cell expression	C179A mutant of hamster PrP with bovine prolactin signal sequence; EGFP @ unique Bsu36I site downstream of signal sequence.	pcDNA5/FRT/TO	CMV Tet-on
pcDNA5-FRT/TO-EGFP-PrP	mammalian cell expression	Wild-type human PrP, with EGFP @ unique Bsu36I site downstream of signal sequence.	pcDNA5/FRT/TO	CMV Tet-on

### Antibodies and recombinant proteins

The following antibodies were used in this study have either been described previously or obtained from sources: custom rabbit antisera raised against recombinant GFP and RFP (Stefanovic and Hegde, 2007); anti-FLAG (M2)-HRP conjugated (Sigma); rabbit anti-TMED10 raised against a C terminal peptide (Satpute-Krishnan et al., 2014); rabbit anti-TMED2, anti-TMED7, anti-TMED9, and anti-TMED10 lumenal domain (gift of Felix Weiland); anti-CNX N-terminus (Enzo ADI-SPA-865); rabbit anti-CNX C terminus (Enzo ADI-SPA-860); rabbit anti tagBFP (evrogen anti-tRFP AB233); mouse anti-AP2 (anti-alpha adaptin, BD Biosciences 610502); mouse anti-Transferrin receptor (Invitrogen 136800); mouse anti-BiP (BD Biosciences 610979). In addition, anti-GFP nanobody was recombinantly expressed in *E. coli*, purified, and labeled with fluorophores as described next.

His-tagged Nanobody-FLAG, Nanobody-FLAG-APEX2, and FKBP*12(F36V) were purified from *E. coli* (BL21) pLysS cells using immobilized metal affinity chromatography. In brief, cells were transformed with the expression plasmids encoding the desired proteins and grown at 37°C in LB under the appropriate antibiotic selection. Expression was induced with 0.1 mM IPTG when bacteria reached an A_600_ of 0.6. Induction proceeded at 16–18°C overnight. Cells were harvested by centrifugation, resuspended in 30 ml ice cold lysis buffer [1X PBS, 300 mM NaCl, 10 mM imidazole, DNase, 1 mM β-mercaptoethanol, 1 mM PMSF, 1 mM Benzamidine, 1X protease inhibitor cocktail (Roche)] per L culture, and lysed by sonication. The lysates were clarified by centrifugation and passed by gravity flow over columns of chelating sepharose (GE Healthcare Life Sciences) charged with Co^2+^. Columns were successively washed with 10 column volumes each of lysis buffer containing 10, 15, and 20 mM imidazole. Proteins were eluted with 200 mM imidazole in 1xPBS and 150 mM NaCl. Peak elutions as judged by A_280_ readings were pooled. In most cases, the proteins were desalted into PBS using a PD-10 column (GE Healthcare). All proteins were supplemented with 10% glycerol prior to storage. To make fluorescently tagged nanobody, 190 nmol of purified nanobody was mixed with 1 mg Alexa Fluor NHS Ester in 50 mM NaHCO_3_ for 1 hr at room temperature, and subsequently separated from free dye by desalting through a PD-10 column equilibrated in PBS.

### Flow cytometry

Cells were resuspended by gentle pipetting in PBS. Unless otherwise indicated, cell surface labeling was performed on ice for 30 min with 200 nM Alexa546- or Alexa647-conjugated anti-GFP Nb in PBS containing 10% FBS. In experiments measuring nanobody internalization, 10 nM Nb was added to the extracellular medium and cells were incubated for the indicated times at 37°C. Total GFP or YFP fluorescence was measured in cells that were not incubated with fluorescent Nb in order to avoid mild fluorescence quenching effects on GFP that were observed upon Nb binding. Cells in both surface labeling and internalization experiments were subsequently washed in PBS and resuspended in 10% FBS with 1 ug/ml DAPI as a viability marker (except when a BFP-conjugated protein was present), and filtered through a 70 μm mesh to remove any clumped cells. Cells were subsequently analyzed on a Beckton Dickinson LSRII or LSRFortessa flow cytometer, and data was analyzed using FlowJo software. A minimum of 10,000 transfected cells were analyzed in each condition. Each experiment is internally controlled and the control cells that are shown in each graph were grown, harvested, and analyzed in parallel with the experimental sample. The numerical values of fluorescence intensity cannot be directly compared across experiments because the absolute number assigned to nanobody fluorescence is dependent on the model of flow cytometer, the settings, and calibration. In Figure 4C, 10^6^ TMED10-transfected cells were selected on the basis of moderate RFP fluorescence using a Sony Biotechnology Synergy High Speed Cell Sorter.

### Fluorescence microscopy

For fixed cell imaging, cells were plated on 13 mm round coverslips (VWR), fixed in 4% formaldehyde, and imaged using a Nikon TE2000 inverted fluorescence microscope using a 100x objective. For live cell imaging, cells were plated on μ-dishes (ibidi) and imaged using an Andor Revolution Spinning Disk inverted microscope, 60x objective. When live cells were imaged over a time course (Figure 1E), dishes were coated with 10 μg/ml fibronectin for 1 hr at 37°C and washed three times with PBS prior to seeding cells, in order to enhance cell adherence. When necessary, image brightness was adjusted by equivalent amounts in all conditions using Fiji software or Adobe Photoshop.

### Sucrose gradient fractionation

Analytical scale 0.2 mL gradients were prepared in 7 × 20 mm centrifuge tubes (Beckman 343775) by successively layering 40 μL of 25%, 20%, 15%, 10%, and 5% sucrose (w/v) in 50 mM HEPES pH 7.4, 150 mM NaCl, 1% CHAPS. Gradients were then allowed to stand for 35–60 min at 4°C. The samples in lysis buffer (50 mM HEPES, 150 mM NaCl, 1% CHAPS) were loaded on top of the gradients and centrifuged in a TLS- 55 rotor at 55,000 rpm for 2 hr 25 min at 4°C with slow acceleration and deceleration. Eleven 20 μL fractions were successively collected from the top and used directly for western blot analysis.

### Immunoprecipitation

In order to isolate the surface-localized population of GFP-PrP and GFP-PrP*, cells were labeled on ice for 1 hr with 200 nM 3xFLAG-tagged anti-GFP Nb, then washed to remove all free Nb. The cells were then sedimented by centrifugation prior to lysis. For both surface and total immunoprecipitation experiments, cells were lysed in 50 mM HEPES, 150 mM NaCl, 1% CHAPS, and EDTA-free protease inhibitor cocktail (Roche). The insoluble material was removed by centrifugation at maximum speed in a microcentrifuge for 10 min at 4°C. The soluble fraction was incubated with anti-FLAG M2 affinity resin (Sigma) for surface IPs, and anti-GFP nanobody conjugated to sepharose beads (Chromotek and homemade) for GFP IPs at 4°C for 1–4 hr. Beads were washed three times in lysis buffer and eluted with 0.1 mg/ml 3x-FLAG peptide or 2x sample buffer (100 mM Tris pH 6.8, 2% SDS, 20% glycerol, 200 mM DTT) prior to further analysis.

### Mass spectrometry

For protein identification in immunoprecipitation experiments, eluates were separated by SDS-PAGE and excised gel bands were washed, reduced with DTT, alkylated with iodoacetamide (IAA) and digested with trypsin in ammonium bicarbonate overnight at 37°C. Extracted peptide mixtures were analyzed by LC/MS/MS in an Orbitrap Velos mass spectrometer (Thermo Fisher Scientific). Quantitative mass spectrometry was performed by on-bead trypsin digestion of samples, followed by tandem mass tag (TMT) labelling. In brief, protein samples on beads were reduced with 10 mM DTT at 56°C for 30 min and alkylated with 15 mM IAA for 30 min in the dark at 22°C. The alkylation reaction was quenched by the addition of DTT and samples were digested with trypsin (Promega) overnight at 37°C. Each supernatant was transferred to a fresh Eppendorf tube, and the beads were extracted once with 50% acetonitrile/0.1% TFA and combined with the corresponding supernatant. The peptide mixtures were then partially dried in a Speed Vac and desalted using C18 (3M Empore) stage tips containing Poros R3 resin (Applied Biosystems). Bound peptides were eluted sequentially with 30%, 50% and 80% acetonitrile in 0.1%TFA and lyophilized. Dried peptide mixtures from each condition were re-suspended in 40 ul of 5% acetonitrile, 200 mM triethyl ammonium bicarbonate. For TMT labelling, 0.8 mg of TMT reagents (Thermo Fisher Scientific) were reconstituted in 41 ul anhydrous acetonitrile. 10–15 ul of TMT was added to each peptide mixture and incubated for 1 hr at room temperature. The labeling reactions were terminated by incubation with 4 ul 5% hydroxylamine for 15 min, and labeled samples were subsequently pooled. A Speed Vac was used to remove acetonitrile, and samples were desalted as above. In the experiment in which cells were subjected to ER stress, samples were additionally fractionated with C18 stage tips using 10 mM ammonium bicarbonate and acetonitrile gradients and acidified. Liquid chromatography was performed on a fully automated Ultimate 3000 RSLC nano System (Thermo Scientific) fitted with a 100 µm x 2 cm PepMap100 C18 nano trap column and a 75 μm × 25 cm reverse phase C18 nano column (Aclaim PepMap, Thermo Scientific). Samples were separated using a binary gradient consisting of buffer A (2% acetonitrile, 0.1% formic acid) and buffer B (80% acetonitrile, 0.1% formic acid). Peptides were eluted at 300 nL/min with an acetonitrile gradient. The HPLC system was coupled to a Q Exactive Plus hybrid quardrupole-Orbitrap mass spectrometer (Thermo Fisher Scientific) equipped with a nanospray ion source. The acquired raw MS/MS files were processed using Proteome Discoverer (version 2.1, Thermo Scientific) or MaxQuant (Cox and Mann, 2008) with the integrated Andromeda search engine.

### Proximity biotinylation

Proximity labeling was performed as described (Hung et al., 2016). In brief, cells were labeled on ice for 30 min with anti-GFP nanobody conjugated to APEX2 at 200 nM (saturating) or 0.5 nM (to achieve sub-saturating labeling of GFP-PrP). Excess nanobody was removed by washing in PBS, and cells were resuspended in 500 μm biotin-phenol (Iris Biotech LS-3500) in PBS at room temperature. Hydrogen peroxide (Sigma) was added to a final concentration of 1 mM for 1 min before quenching with 10 mM sodium ascorbate (Sigma), 5 mM Trolox (Acros), and 10 mM sodium azide (VWR). Cells were washed three times in PBS containing the quenching reagents, pelleted, and lysed as above. Biotinylated proteins were detected by western blotting using HRP-conjugated streptavidin (ThermoFisher 43–4323). For isolation of biotinylated proteins, cells were lysed in 50 mM HEPES, 150 mM NaCl, 2 mM MgAc, and 1% CHAPS. Lysates were desalted using a Sephadex G-25 column to remove excess biotin-phenol, and incubated with Streptavidin T1 Dynabeads (Invitrogen) overnight at 4°C. Beads were subjected to a series of stringent washes in lysis buffer; 1% SDS in 10 mM HEPES; 1 M KCl in 10 mM HEPES with 0.5% CHAPS; and 2 M urea in 10 mM Tris with 0.5% CHAPS. Proteins were eluted in sample buffer containing 2 mM biotin.

### Drug treatments

Drugs were used at the following concentrations: thapsigargin (Sigma) at 100 nM, Bafilomycin A1 (Sigma) at 250 nM, Brefeldin A (Invitrogen) at either 2.5 μg/ml or 1 μM as indicated, Shield1 (Clontech/Takara) at 2 uM, and Cycloheximide (Sigma) at 100 μg/ml.

## Data Availability

All data generated or analyzed during this study are included in the manuscript and supporting files. Source data has been provided for Figure 5E.

## Additional files

Transparent reporting form
