# Supplementary material for: Misfolded GPI-anchored proteins are escorted through the secretory pathway by ER-derived factors
Source: eLife. 2019 May 16;8:e46740. doi: 10.7554/eLife.46740 (PMC6541436; doi:10.7554/eLife.46740)
Supplement: Peer review history

# Decision letter

**DOI:** 10.7554/eLife.46740.020

**Affiliations:** Weizmann InstituteIsrael; University of SevilleSpain

In the interests of transparency, eLife includes the editorial decision letter and accompanying author responses. A lightly edited version of the letter sent to the authors after peer review is shown, indicating the most substantive concerns; minor comments are not usually included.

Thank you for submitting your article "Misfolded GPI-anchored proteins are escorted through the secretory pathway by ER-derived factors" for consideration by *eLife*. Your article has been reviewed by three peer reviewers, and the evaluation has been overseen by a Reviewing Editor and David Ron as the Senior Editor. The following individual involved in review of your submission has agreed to reveal their identity: Veit Goder (Reviewer #2).

The reviewers have discussed the reviews with one another and the Reviewing Editor has drafted this decision to help you prepare a revised submission.

Please find below the summary of the paper and the main points that we would like you to address before resubmission.

The presented work addresses the cellular mechanisms that operate in post-ER protein quality control. Using mammalian cell culture as a model system, the authors investigate how misfolded GPI-anchored proteins (GPI-APs) are routed to the lysosome for degradation. Surprisingly, they show that the entire population of misfolded GPI-APs is first routed to the cell surface. This is followed by rapid internalization via endocytosis and routing to the lysosome. The physiological relevance for this "extended" traffic from the ER to the lysosome via the cell surface remains unclear, but the authors provide some interesting theories in the Discussion section.

Most relevant, however, are their findings that several ER chaperones and trafficking receptors remain bound to misfolded GPI-APs all along their way to the cell surface. This is a new concept and provides insight into novel cellular mechanisms. The interactions with ER chaperones and receptors are specific for misfolded GPI-APs; a correctly folded species is not associated with chaperones and only drastically less with trafficking receptors after ER exit. Moreover, the presence of chaperones and trafficking factors in complex with misfolded GPI-APs at the cell surface is crucial for their endocytosis and subsequent degradation. Thus, some "post-ER quality control mechanisms" for a subclass of proteins of the secretory pathways involve in fact ER chaperones.

The work is outstanding, the experimental approaches are original and the data are solid. This work will be highly suitable for *eLife* with only minor changes or additions requested below:

Experimental additions:

1) In Figure 1D the authors show complete degradation by 3 hours of PrP*-GFP in the presence of CHX but in Figure 1E the time lapse only continues until 2 hours (120 minutes) with no CHX. It would be important to see the microscopy that correlates with the flow cytometry of Figure 1D (time points until 3 hours +CHX) to ensure that there is no change in trafficking pattern with CHX or at longer times.

2) Figure 6D exploits the high on rates of the RFP nanobodies for RFP, such that RFP-TMED10 molecules at the cell surface pick up the nanobody, internalise it and the fluorescent label of the nanobody accumulates in the cell and generates a FACS signal. Could the authors expand on the client dependence of this process: Was it enhanced by co-expression of PrP*? By exposure of the cells to thapsigargin? Was it inhibited by brefeldin A? Have the authors tried to detect PrP*/thapsigargin dependent exposure of BiP or CNX on the cell surface by FACS?

The claim that PrP* is accompanied by ER resident proteins to the cell surface is one of this paper's most important. Right now the strongest support of this is the RFP-TMED10 expression on the cell surface however in this experiment TMED10 is overexpressed and this can lead to abnormal trafficking of this protein. Hence, any evidence to support further the notion of ER chaperones traveling with clients all the way to the cell surface on their way to degradation would greatly strengthen the manuscript.

Textual changes:

1) In the Introduction when PrP is first introduced the authors may want to already mention that PrP is a model GPI anchor protein.

2) In the first Results section the authors write GFP-wtPrP – this "wt" denotation is not required nor is it used elsewhere.

3) In Figure 3—figure supplement 1E – it would be helpful to the readers to label on the images that they are KO of TMED10.

4) Figure 5B – it would be helpful to readers if it is labeled on the panel itself that KO refers to KO of TMED10

5) In Figure 4D the authors arrive to some conclusions about requirements of various TMED family members for PrP* trafficking based on siRNA knockdown experiments. However, since the non-requirement of some TMEDs is discussed based on the absence of phenotype, the authors should show the effectiveness of knockdown (or wait with the conclusion until showing the supporting interactome data in the next figure).

6) Figure 4—figure supplement 1A and Figure 6 for all sucrose gradients, a MW standard would be helpful.

7) Figure 5F – The extended interaction of TMED10 also with GFP-wtPrP: could that be due to the GFP moiety on wtPrP, (slow folding?), can this be checked with wtPrP without a tag?

8) In Figure 2E to G, the surface levels of GFP-PrP* were analyzed by AP2 knockdown, expression of Dynamin or brefeldin A treatment. However, since the Nb fluorescence levels are very different between Figure 2E, F and G, it is difficult to judge the results. For example, the fluorescence levels of AP2-KD in Figure 2E is similar to those of control in Figure 2F and G. Please double check this and provide more representative images if possible or just double check the reproducibility.

9) In Figure 6C, the authors showed that surface PrP* associates with calnexin and TMED10. As the control, they used PrP and loaded only 5% of the IP fraction. Please elaborate on whether, even if the authors load 100% wild-type PrP samples, the calnexin and TMED10 are not detected in the IP fraction?

10) Similarly, in Figure 6F, did the authors load same amounts of samples in the strep PD fraction? If so, pull downed Nb-APEX was the same level between PrP and PrP* samples, even if the surface expressions of PrP and PrP* were quite different. Is it correct?

11) In Figure 6D, the authors analyzed the internalization of anti-RFP nanobody in RFP-HA-TMED10-expressing cells. It is possible that RFP-HA-TMED10 leaks to the cell surface due to the overexpression. Do the authors maybe have fluorescent microscopy images to verify the cellular localization of RFP-HA-TMED10 and nanobody signals?.

12) In the last paragraph of the subsection “A complex of p24 proteins is required for PrP* trafficking and degradation”, the authors showed that knockdowns of TMED10, TMED2, TMED5 and TMED9 were impaired the PrP* traffic. The same combination of TMED members has been reported for general transport of GPI-anchored proteins from the ER (J Cell Biol. (2011) 194(1):61-75), suggesting that the TMED subfamily members are utilized not only for exit of misfolded GPI-anchored proteins from the ER, but also for that of folded GPI-anchored proteins. Please refer to it in the manuscript.

13) Subsection “Resident ER proteins are associated with cell surface PrP*”, first paragraph, (Figure 6B, bottom panels) should be Figure 6A.

Two points were also raised as suggestions (we do *not* see them as essential for submitting a revised manuscript):

1) In Figure 7I wonder whether surface-exposed FKBP*-YFP-GPI after Shield1 washout will be degraded if soluble chaperones (or soluble portions of membrane-bound chaperones) are supplied to the medium (i.e. if the presence of chaperones would suffice to trigger degradation)?

2) In Figure 7, FKBP*-YFP-GPI was used to show the stability on the cell surface. The reviewer recommends to check whether the FKBP*-YFP-GPI is also expressed on the cell surface by ER stress using thapsigargin treatment, similar to PrP*.

# Reply: Author response

**DOI:** 10.7554/eLife.46740.021

Experimental additions:

1) In Figure 1D the authors show complete degradation by 3 hours of PrP*-GFP in the presence of CHX but in Figure 1E the time lapse only continues until 2 hours (120minutes) with no CHX. It would be important to see the microscopy that correlates with the flow cytometry of Figure 1D (time points until 3 hours +CHX) to ensure that there is no change in trafficking pattern with CHX or at longer times.

As suggested, we have carried out the time lapse microscopy for 3 h in the presence of cycloheximide (CHX) to illustrate that trafficking is not affected, and that overall GFP signal decreases consistent with the flow cytometry experiment. The new experiment is shown in Figure 1E, and the previous experiment has now moved to Figure 3—figure supplement 1, where it can be compared to the analogous experiment in TMED10 knockout cells.

2) Figure 6D exploits the high on rates of the RFP nanobodies for RFP, such that RFP-TMED10 molecules at the cell surface pick up the nanobody, internalise it and the fluorescent label of the nanobody accumulates in the cell and generates a FACS signal. Could the authors expand on the client dependence of this process: Was it enhanced by co-expression of PrP*? By exposure of the cells to thapsigargin? Was it inhibited by brefeldin A? Have the authors tried to detect PrP*/thapsigargin dependent exposure of BiP or CNX on the cell surface by FACS?

The claim that PrP* is accompanied by ER resident proteins to the cell surface is one of this paper's most important. Right now the strongest support of this is the RFP-TMED10 expression on the cell surface however in this experiment TMED10 is overexpressed and this can lead to abnormal trafficking of this protein. Hence, any evidence to support further the notion of ER chaperones traveling with clients all the way to the cell surface on their way to degradation would greatly strengthen the manuscript.

We agree with the reviewers that the discovery of ER resident proteins accompanying a misfolded client to the cell surface is the most significant finding of our paper. This conclusion is supported by three independent results using orthogonal approaches: (i) time-resolved analysis of GFP-PrP* interactions during acute stress shows little or no change in association with endogenous Calnexin and TMED10 (Figure 5D, 5F; Figure 5—figure supplement 1C) despite complete egress from the ER (Figure 1E; Figure 3—figure supplement 1E) and near-quantitative transit through the cell surface (Figure 3B) over this time frame; (ii) selective immunopurification of surface-localised GFP-PrP* co-purifies endogenous Calnexin and TMED10 (Figure 6B, 6C; Figure 6—figure supplement 1A-1D); (iii) proximity labelling selectively of surface GFP-PrP* in intact cells labels Calnexin (Figure 6F). In each of these experiments, selectivity is observed for PrP* over wild type PrP. Therefore, the evidence strongly supports our conclusion that misfolded PrP* engages ER proteins that retain their interactions all the way to the plasma membrane; we cannot think of an alternative model that matches all of the above results, and none was offered by the reviewers.

The above experiments use GFP-PrP* (i.e., the substrate) as the handle with which to detect and analyze interacting partners. As rightly pointed out by the reviewers, the ‘reverse’ experiment of analyzing the interacting partners would be a nice complement to the existing evidence. While one can show that native IPs of TMED10 or calnexin by can co-precipitate PrP*, the challenge is to analyze ONLY the surface population. We have made several attempts toward this goal, but have faced substantial challenges due to the extremely low proportion of these normally ER-resident proteins on the surface combined with the limitations of available antibodies. As discussed in the text and documented in Figure 6C (and Figure 6—figure supplement 1D), only ~0.5% of cellular Calnexin and TMED10 is engaged with PrP* on the surface. Monitoring this tiny population requires reagents that bind with exquisitely high affinity and specificity, such as the anti-GFP nanobody that enabled us to detect surface-localised GFP-PrP*. Unfortunately, labelling with available antibodies against the non-cytosolic domains of endogenous ER resident proteins (Calnexin, TMED10, and BiP) has not provided sufficient signal-to-noise to allow for direct observation of these factors at the surface.

This reagent limitation is why we turned to exogenous RFP-TMED10 (to exploit the high affinity anti-RFP nanobody) expressed in TMED10-knockout cells. As correctly noted by the reviewers, this approach suffers from the potential for overexpression artefacts. In addition to the caveat of possible mislocalisation, overexpression of an ER protein can, in itself, be an ER stressor (Satpute-Krishnan et al., 2014). Thus, under these conditions, cells show a strongly blunted response to additional ER stressors, confounding experiments to analyze stress-dependence of surface RFP-TMED10. We have concluded that to properly monitor TMED10, Calnexin, and other factors, it will be necessary to tag the factor in the genome (so that high affinity nanobodies can be exploited as we did with GFP-PrP*), verify that the tag does not disrupt either function or trafficking, then perform the analysis. This will be an extensive endeavour for future studies. The anti-RFP Nb uptake by RFP-TMED10 was intended as an initial step toward this aim. Given the caveats of this experiment (and the already strong support for the central conclusion as summarized above), we have opted to remove this experiment, and point out the above limitations in the Discussion (fifth paragraph).

Textual changes:

1) In the Introduction when PrP is first introduced the authors may want to already mention that PrP is a model GPI anchor protein.

We have made this change (Introduction, second paragraph).

2) In the first Results section the authors write GFP-wtPrP – this "wt" denotation is not required nor is it used elsewhere.

We thank the reviewers for spotting this, and have made the appropriate change.

3) In Figure 3—figure supplement 1E – it would be helpful to the readers to label on the images that they are KO of TMED10

We have made this change.

4) Figure 5B – it would be helpful to readers if it is labeled on the panel itself that KO refers to KO of TMED10

We have made this change.

5) In Figure 4D the authors arrive to some conclusions about requirements of various TMED family members for PrP* trafficking based on siRNA knockdown experiments. However, since the non-requirement of some TMEDs is discussed based on the absence of phenotype, the authors should show the effectiveness of knockdown (or wait with the conclusion until showing the supporting interactome data in the next figure).

Because we do not have suitable antibodies to verify all the knockdowns, we have removed the table in Figure 4D and tempered our conclusion in the text about which TMEDs are involved in PrP* trafficking (subsection “A complex of p24 proteins is required for PrP* trafficking and degradation”, last paragraph). Instead, we simply document that not all TMEDs impact PrP* trafficking by showing that TMED7 knockdown, verified by immunoblotting, does not have an effect on PrP* (Figure 4—figure supplement 1E).

6) Figure 4—figure supplement 1A and Figure 6 for all sucrose gradients, a MW standard would be helpful.

This has been added.

7) Figure 5F – The extended interaction of TMED10 also with GFP-wtPrP: could that be due to the GFP moiety on wtPrP, (slow folding?), can this be checked with wtPrP without a tag?

In Figure 5F, total GFP-PrP was immunoprecipitated using the anti-GFP nanobody, whose binding is strictly dependent on folded GFP. Therefore, we don’t think TMED10 association via misfolded GFP is a likely explanation.

8) In Figure 2E to G, the surface levels of GFP-PrP* were analyzed by AP2 knockdown, expression of Dynamin or brefeldin A treatment. However, since the Nb fluorescence levels are very different between Figure 2E, F and G, it is difficult to judge the results. For example, the fluorescence levels of AP2-KD in Figure 2E is similar to those of control in Figure 2F and G. Please double check this and provide more representative images if possible or just double check the reproducibility.

The experiments in Figure 2E-G are independent experiments performed on different days. Each one is internally controlled and the control cells that are shown in each graph were grown, harvested, and analyzed in parallel with the experimental sample. The numerical values from different experiments are not comparable to each other because the absolute number assigned to nanobody fluorescence is dependent on the model of flow cytometer (we have several), the settings, and calibration. This is why all flow cytometry experiments throughout the study always have the respective controls analyzed at the same time. We have made a note in the Materials and methods section to clarify this point (subsection “Flow cytometry”).

9) In Figure 6C, the authors showed that surface PrP* associates with calnexin and TMED10. As the control, they used PrP and loaded only 5% of the IP fraction. Please elaborate on whether, even if the authors load 100% wild-type PrP samples, the calnexin and TMED10 are not detected in the IP fraction?

The reviewer’s concern about loading only 5% of the PrP IP fraction is well taken, so we repeated the experiment in another way. We titrated the amount of nanobody used to label PrP cells in order to achieve the same amount of surface labelling and recovery as PrP* cells. Consequently, we were able to isolate equal amounts of surface-localized PrP and PrP*, and demonstrate that calnexin and TMED10 preferentially associate with PrP*. The approach is explained in detail in the legend for Figure 6C.

If all of the surface PrP and PrP* is isolated from cells, one recovers ~20-fold more PrP than PrP* (e.g., Figure 6—figure supplement 1A and 1B). Even under these conditions, the PrP sample contains less calnexin (see stained gels in Figure 6—figure supplement 1A and 1B) and similar amounts of TMED10 (not shown) as the PrP* sample. Thus, the ratio of associating factor to PrP* at the cell surface is 20-50 times higher than for PrP. The small proportion of PrP that associates with Calnexin and TMED10 at the surface might be the population that fails to fold during biogenesis at the ER, thereby necessitating sustained interaction with the ER-derived factors as with PrP*.

10) Similarly, in Figure 6F, did the authors load same amounts of samples in the strep PD fraction? If so, pull downed Nb-APEX was the same level between PrP and PrP* samples, even if the surface expressions of PrP and PrP* were quite different. Is it correct?

In Figure 6F, the Nb-APEX was titrated in the PrP condition to achieve equal cell surface labelling to that in the PrP* condition (as described in the response to the previous comment). This allowed us to examine the neighbouring proteins of an equivalent number of surface APEX molecules on the surface of PrP and PrP* cells. This is why equal amounts of Nb-APEX was recovered when equal amounts of sample are analyzed on the blot. This is stated in the legend.

11) In Figure 6D, the authors analyzed the internalization of anti-RFP nanobody in RFP-HA-TMED10-expressing cells. It is possible that RFP-HA-TMED10 leaks to the cell surface due to the overexpression. Do the authors maybe have fluorescent microscopy images to verify the cellular localization of RFP-HA-TMED10 and nanobody signals?.

In view of the reviewers’ concerns about overexpression of exogenous RFP-TMED10, we have opted to omit Figure 6D until better reagents are available (see detailed reply above).

12) In the last paragraph of the subsection “A complex of p24 proteins is required for PrP* trafficking and degradation”, the authors showed that knockdowns of TMED10, TMED2, TMED5 and TMED9 were impaired the PrP* traffic. The same combination of TMED members has been reported for general transport of GPI-anchored proteins from the ER (J Cell Biol. (2011) 194(1):61-75), suggesting that the TMED subfamily members are utilized not only for exit of misfolded GPI-anchored proteins from the ER, but also for that of folded GPI-anchored proteins. Please refer to it in the manuscript.

We thank the reviewers for their observation and included this reference.

13) Subsection “Resident ER proteins are associated with cell surface PrP*”, first paragraph, (Figure 6B, bottom panels) should be Figure 6A.

We have made this change.

Two points were also raised as suggestions (we do not see them as essential for submitting a revised manuscript):

1) In Figure 7I wonder whether surface-exposed FKBP*-YFP-GPI after Shield1 washout will be degraded if soluble chaperones (or soluble portions of membrane-bound chaperones) are supplied to the medium (i.e. if the presence of chaperones would suffice to trigger degradation)?

This is a very interesting suggestion, and one we have considered trying. However, we believe there is still a requirement for some sort of transmembrane factor in order to “communicate” with endocytosis machinery in the cytosol.

2) In Figure 7, FKBP*-YFP-GPI was used to show the stability on the cell surface. The reviewer recommends to check whether the FKBP*-YFP-GPI is also expressed on the cell surface by ER stress using thapsigargin treatment, similar to PrP*.

Figure 7—figure supplement 1A shows extracellular nanobody uptake before (time 0) and after (later time points) thapsigargin treatment without Shield 1. Time 0 represents surface staining because no uptake has been allowed to happen. Similar to PrP*, thapsigargin promotes nanobody uptake in the absence of Shield1, consistent with FKBP*-YFP-GPI exiting from the ER and transiently transiting the cell surface en route to degradation. Thus, FKBP*-YFP-GFP goes to the surface upon thapsigargin treatment similar to PrP*.
